# Temporal trends in incidence, patient characteristics, microbiology and in-hospital mortality in patients with infective endocarditis: a contemporary analysis of 86,469 cases between 2007 and 2019

**DOI:** 10.1007/s00392-022-02100-4

**Published:** 2022-09-12

**Authors:** Peter Moritz Becher, Alina Goßling, Nina Fluschnik, Benedikt Schrage, Moritz Seiffert, Niklas Schofer, Stefan Blankenberg, Paulus Kirchhof, Dirk Westermann, Daniel Kalbacher

**Affiliations:** 1 Department of Cardiology, University Medical Center Hamburg-Eppendorf, University Heart & Vascular Center Hamburg, Martinistrasse 52, 20246 Hamburg, Germany; 2 German Center of Cardiovascular Research (DZHK), Partner Site Hamburg/Kiel/Lübeck, Hamburg, Germany; 3 Department of Cardiology and Angiology I, Medical Faculty, University Heart Center Freiburg, Bad Krozingen, University of Freiburg, Freiburg, Germany

**Keywords:** Infective endocarditis, Incidence, Valve, Outcomes, Trends, Mortality

## Abstract

**Background:**

Infective endocarditis (IE) is characterized by high morbidity and mortality rates, despite recent improvements in diagnostics and treatment. We aimed to investigate incidence, clinical characteristics, and in-hospital mortality in a large-scale nationwide cohort.

**Methods:**

Using data from the German Federal Bureau of Statistics, all IE cases in Germany between 2007 and 2019 were analyzed. Logistic regression models were fitted to assess associations between clinical factors and in-hospital mortality.

**Results:**

In total, 86,469 patients were hospitalized with IE between 2007 and 2019. The mean age was 66.5 ± 14.7 years and 31.8% (*n* = 27,534/86,469) were female. Cardiovascular (CV) comorbidities were common. The incidence of IE in the German population increased from 6.3/100,000 to 10.2/100,000 between 2007 and 2019. *Staphylococcus* (*n* = 17,673/86,469; 20.4%) and *streptococcu*s (*n* = 17,618/86,469; 20.4%) were the most common IE-causing bacteria. The prevalence of *staphylococcus* gradually increased over time, whereas blood culture-negative IE (BCNIE) cases decreased. In-hospital mortality in patients with IE was 14.9%. Compared to BCNIE, *staphylococcus* and Gram-negative pathogens were associated with higher in-hospital mortality. In multivariable analysis, factors associated with higher likelihood of in-hospital mortality were advanced age, female sex, CV comorbidities (e.g., heart failure, COPD, diabetes, stroke), need for dialysis or invasive ventilation, and sepsis.

**Conclusions:**

In this contemporary cohort, incidence of IE increased over time and in-hospital mortality remained high (~ 15%). While *staphylococcus* and *streptococcus* were the predominant microorganisms, bacteremia with *staphylococcus* and Gram-negative pathogens were associated with higher likelihood of in-hospital mortality. Our results highlight the need for new preventive strategies and interventions in patients with IE.

**Graphical abstract:**

**Figure:**
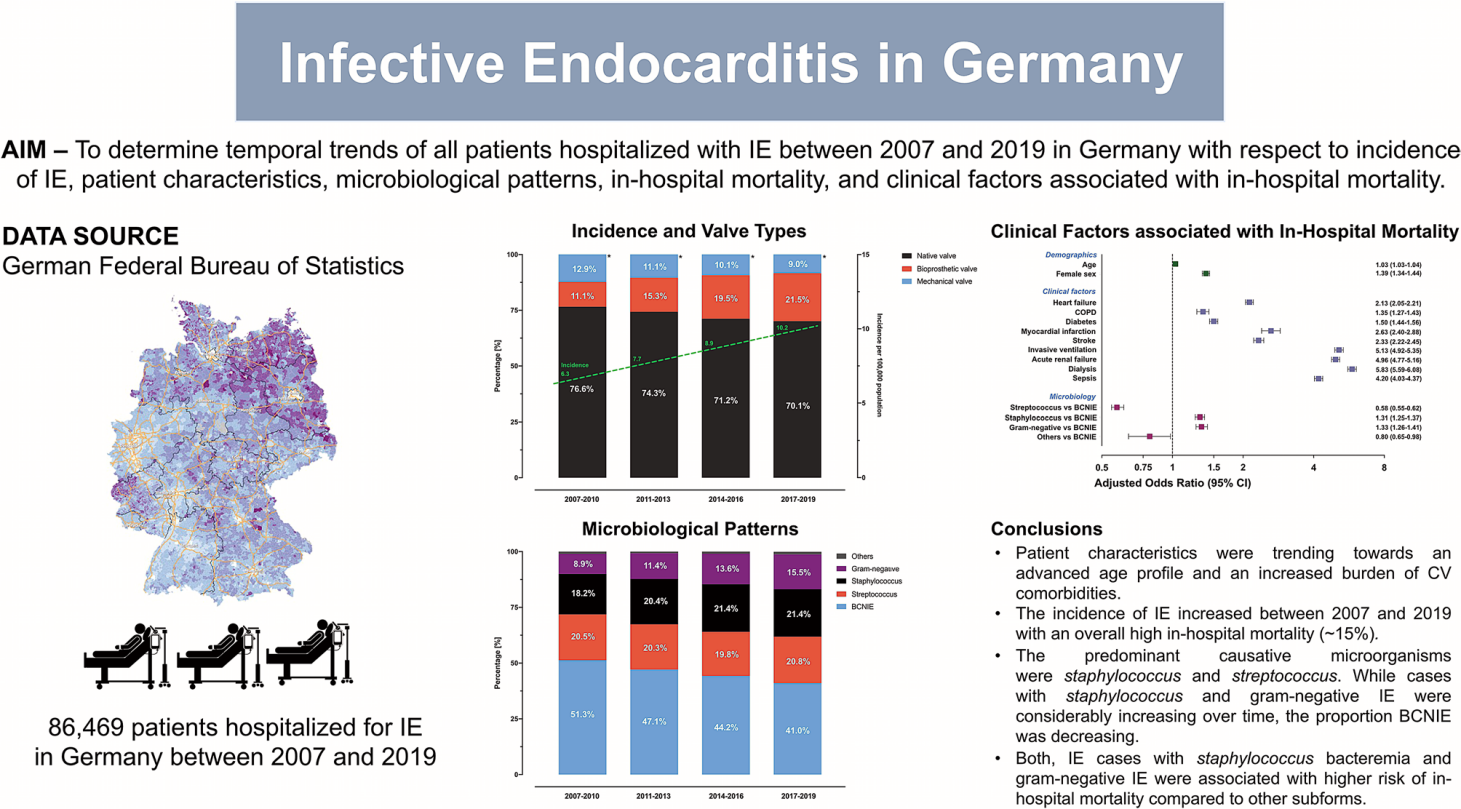
Infective endocarditis in Germany. *BCNIE* blood culture-negative infective endocarditis, *IE* infective endocarditis

**Supplementary Information:**

The online version contains supplementary material available at 10.1007/s00392-022-02100-4.

## Introduction

Infective endocarditis (IE) is a worldwide occurring disease associated with high morbidity including substantially reduced quality of life, high risk of reinfection and prolonged hospitalization as well as high mortality rates ranging from 14 to 22% [1–3].

Although IE is relatively infrequent (reported incidence 3–10 per 100,000 population), it is causing severe morbidity and was responsible for about 1.6 million disability-adjusted life years in 2010 alone [4–7].

New antimicrobial therapies, early surgery, and better intensive care improved treatment of IE [7]. At the same time, a growing number of older patients with many cardiovascular (CV) comorbidities receive cardiac valve or device implants and resistance to antimicrobial therapies is increasing, creating a growing population at high risk for IE [8–11].

Therefore, the aim of this study was to describe temporal trends in a nationwide sample of all patients hospitalized with IE between 2007 and 2019 in Germany with respect to (1) incidence of IE; (2) patient characteristics; (3) microbiological patterns; (4) in-hospital mortality; and (5) clinical factors associated with in-hospital mortality.

## Methods

### Study population and setting

The Federal Bureau of Statistics in Germany collects nationwide administrative data on hospitalized patients. Within this database, all diagnoses and performed procedures are stored. Data collection is mandatory for all hospitals owing to regulatory requirements in Germany. Diagnoses are coded using the German modification of the International Statistical Classification of Diseases and Related Health Problems, 10th revision (ICD-10-GM); procedures are coded using the German Operational and Procedural codes (OPS).

**Fig. 1:**
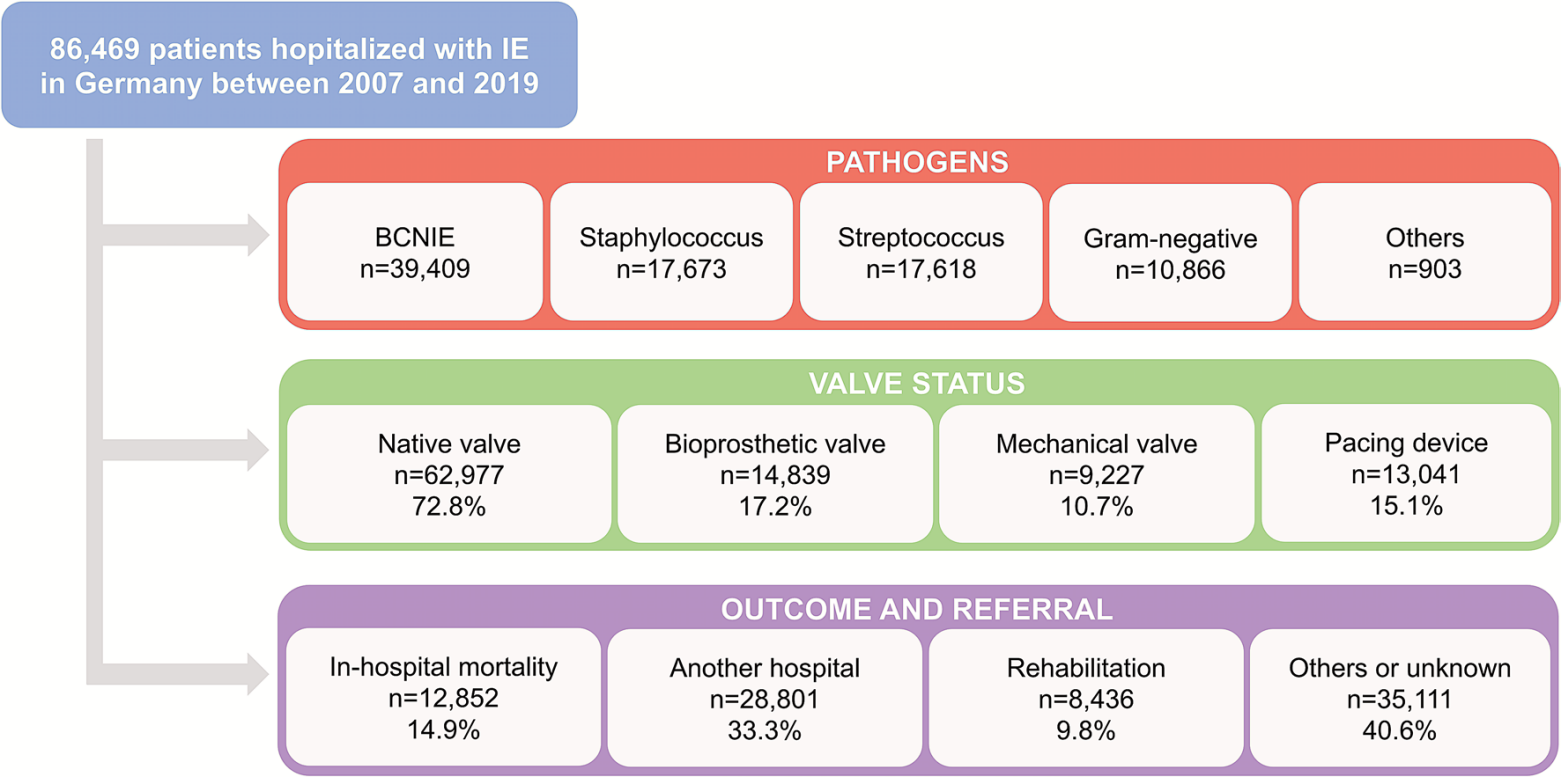
Flowchart of the study population. *BCNIE* blood culture-negative infective endocarditis, *CI* confidence interval, *COPD* chronic obstructive pulmonary disease, *IE* infective endocarditis

For the current analysis, all patients hospitalized with a primary diagnosis of IE (ICD-10-GM code I33, I38–I39) between 2007 and 2019 were considered. Flowchart of patients included in this study is shown in Fig. 1. Patients < 18 years were excluded from the analysis. Data on coexisting conditions, microbiological patterns, treatments, and outcomes were obtained via ICD-10-GM codes or OPS codes (all definitions used for data query are shown in Supplementary Table S1). The subgroup termed “gram-negative” includes both the HACEK pathogens (haemophilus species, aggregate bacter actinomycetemcomitans, cardiobacterium hominis, eikenella corrodens, kingella species) as well as all non-HACEK pathogens, e.g., *enterobacteriaceae* and *pseudomonas species*. The group “others” consists of rare pathogen organisms, which are listed in the Supplementary Table S1. If multiple pathogens were detected, the following hierarchical order was used (others > gram-negative > staphylococcus > streptococcus > blood culture-negative IE [BCNIE]).

This study was performed in accordance with the Declaration of Helsinki. The investigators did not have direct access to individual patient data, but access to fully anonymous summary results from the research data center. All data have been routinely collected during clinical practice, therefore, German law did not require institutional review board approval and informed consent.

### Statistical analysis

The research data center of the Federal Bureau of Statistics in Germany handled the data export on our behalf. Aggregated statistical analyses were performed using R codes sent by the authors to the research data center. Aggregate data were checked for consistency and prepared for publication.

Binary variables are shown as absolute numbers and percentages, whereas continuous variables are shown as mean ± standard deviation (SD). For between-group comparisons, the Krukal–Wallis test was used for continuous variables and the $\chi^{2}$ test for binary variables. The overall population was separated by year of presentation as follows: 2007–2010, 2011–2013, 2014–2016, 2017–2019. Annual incidence of IE was calculated based on the date of admission to hospital between January 1st and December 31st of each year divided by the population of Germany in that year.

Multivariable logistic regression models were fitted to investigate the association of patient characteristics, clinical factors, microbiological patterns, and treatments with in-hospital mortality. These models were adjusted for age, sex and length of hospital stay.

All statistical analyses were programmed in R statistical software (R Foundation, version 3.5.2, Vienna, Austria) and performed at the Federal Bureau of Statistics in Wiesbaden. A *p* value < 0.05 was considered as statistically significant.

## Results

Between January 1st, 2007 and December 31th, 2019, a total of 86,469 hospitalized cases with the primary diagnosis of IE were registered in Germany. The mean age was 66.5 (± 14.7) years and 27,534 (31.8%) patients were female (Graphical abstract, Table 1).

**Table 1:** Baseline characteristics in patients with infective endocarditis and stratified by microbiology between 2007 and 2019

Variables	Overall cohort	Streptococcus	Staphylococcus	Gram-negative	BCNIE	Others	*p*-value
	(*n* = 86,469)	(*n* = 17,618)	(*n* = 17,673)	(*n* = 10,866)	(*n* = 39,409)	(*n* = 903)	
Demographics							
Age (years)	66.5 ± 14.7	66.3 ± 14.5	65.6 ± 15.4	69.9 ± 13.2	66.2 ± 14.8	65.7 ± 15.1	< 0.001
Sex, female	27,534 (31.8%)	4273 (24.3%)	5557 (31.4%)	4431 (40.8%)	12,996 (33.0%)	277 (30.7%)	< 0.001
Length of hospital stay	23.9 ± 18.3	24.9 ± 16.4	24.9 ± 18.3	32.4 ± 24.0	20.7 ± 16.3	27.5 ± 19.7	< 0.001
Comorbidities							
Hypertension	38,792 (44.9%)	8094 (45.9%)	7868 (44.5%)	5190 (47.8%)	17,246 (43.8%)	394 (43.6%)	< 0.001
Diabetes	23,556 (27.2%)	4273 (24.3%)	5246 (29.7%)	3884 (35.7%)	9914 (25.2%)	239 (26.5%)	< 0.001
COPD	7576 (8.8%)	1405 (8.0%)	1597 (9.0%)	1218 (11.2%)	3288 (8.3%)	68 (7.5%)	< 0.001
PAH	6709 (7.8%)	1377 (7.8%)	1510 (8.5%)	1048 (9.6%)	2701 (6.9%)	73 (8.1%)	< 0.001
History of Stroke	7137 (8.3%)	1261 (7.2%)	1844 (10.4%)	1035 (9.5%)	2924 (7.4%)	73 (8.1%)	< 0.001
Atrial fibrillation	32,955 (38.1%)	6601 (37.5%)	7040 (39.8%)	4949 (45.6%)	14,023 (35.6%)	342 (37.9%)	< 0.001
Hypercholesterinemia	19,319 (22.3%)	4118 (23.4%)	3866 (21.9%)	2786 (25.6%)	8339 (21.2%)	210 (23.3%)	< 0.001
Peripheral vascular disease	5126 (5.9%)	748 (4.3%)	1363 (7.7%)	1006 (9.3%)	1957 (5.0%)	52 (5.8%)	< 0.001
Chronic kidney disease	29,116 (33.7%)	5220 (29.6%)	6391 (36.2%)	4640 (42.7%)	12,591 (32.0%)	274 (30.3%)	< 0.001
Heart failure	34,233 (39.6%)	6571 (37.3%)	7383 (41.8%)	5163 (47.5%)	14,741 (37.4%)	375 (41.5%)	< 0.001
History of bypass surgery	7130 (8.6%)	726 (4.1%)	1551 (8.8%)	999 (9.2%)	3040 (7.7%)	67 (7.4%)	< 0.001
Cardiac valve status							
Mechanical prothesis	9226 (10.7%)	1940 (11.0%)	1720 (9.7%)	1091 (10.0%)	4353 (11.1%)	122 (13.5%)	< 0.001
Biological prothesis	14,839 (17.2%)	3535 (20.1%)	2750 (15.6%)	2043 (18.8%)	6363 (16.2%)	148 (16.4%)	< 0.001
Native valve	62,977 (72.8%)	12,279 (70.0%)	13,321 (75.4%)	7828 (72.0%)	28,907 (73.4%)	642 (71.1%)	< 0.001
Implanted pacing device	13,041 (15.1%)	2303 (13.1%)	3180 (18.0%)	1846 (17.0%)	5597 (14.2%)	115 (12.7%)	< 0.001
Change or extraction of pacing device	3388 (25.9%)	408 (17.4%)	1190 (37.4%)	501 (27.1%)	1248 (22.2%)	41 (35.6%)	< 0.001
Malignancy	4289 (5.0%)	751 (4.3%)	891 (5.0%)	632 (5.8%)	1972 (5.0%)	43 (4.8%)	< 0.001
Immunosuppression	303 (0.4%)	53 (0.3%)	60 (0.3%)	53 (0.5%)	133 (0.3%)	4 (0.4%)	0.108
Clinical presentation							
Myocardial infarction	2312 (2.7%)	364 (2.1%)	493 (2.8%)	375 (3.5%)	1063 (2.7%)	17 (1.9%)	< 0.001
Myocarditis	115 (0.1%)	21 (0.1%)	20 (0.1%)	23 (0.2%)	51 (0.1%)	0.0 (%)	0.132
Severe pulmonary embolism	249 (0.3%)	27 (0.2%)	68 (0.4%)	37 (0.3%)	115 (0.3%)	0.0 (%)	< 0.001
Acute renal failure	17,700 (20.5%)	2797 (15.9%)	4718 (26.7%)	3192 (29.4%)	6804 (17.3%)	189 (20.9%)	< 0.001
Stroke	9810 (11.4%)	1570 (8.9%)	2488 (14.1%)	1443 (13.3%)	3997 (10.1%)	97 (10.7%)	< 0.001
Ischemic	8713 (10.1%)	1570 (8.9%)	2176 (12.3%)	1292 (11.9%)	3587 (9.1%)	88 (9.8%)	< 0.001
Hemorrhagic	1687 (1.9%)	299 (1.7%)	512 (2.9%)	241 (2.2%)	618 (1.6%)	17 (1.9%)	< 0.001
Sepsis	17,463 (20.2%)	1065 (6.0%)	5703 (32.3%)	3463 (31.9%)	7039 (17.9%)	193 (21.4%)	< 0.001
Septic shock	3927 (4.5%)	443 (2.5%)	1154 (6.5%)	805 (7.4%)	1488 (3.8%)	37 (4.1%)	< 0.001
Microbiology							
*Streptococcus*	17,618 (20.4%)	17,618 (100%)	0 (%)	0 (%)	0 (%)	0 (%)	< 0.001
*Staphylococcus*	17,673 (20.4%)	0 (%)	17,673 (100%)	0 (%)	0 (%)	0 (%)	< 0.001
Gram-negative	10,866 (12.6%)	0 (%)	0 (%)	10,866 (100%)	0 (%)	0 (%)	< 0.001
BCNIE	39,409 (45.6%)	0 (%)	0 (%)	0 (%)	39,409 (100%)	0 (%)	< 0.001
Others	903 (1.0%)	0 (%)	0 (%)	0 (%)	0 (%)	903 (100%)	< 0.001
Treatments							
Transoesophageal echocardiography	63,238 (73.1%)	13,470 (76.4%)	13,435 (76.0%)	8599 (79.1%)	27,054 (68.6%)	680 (75.3%)	< 0.001
Invasive ventilation	14,232 (16.5%)	2312 (13.1%)	3634 (20.6%)	2459 (22.6%)	5690 (14.4%)	137 (15.2%)	< 0.001
Non-invasive ventilation	5664 (6.6%)	1005 (5.7%)	1436 (8.1%)	1104 (10.2%)	2054 (5.2%)	65 (7.2%)	< 0.001
Dialysis	12,827 (14.8%)	1822 (10.3%)	3510 (19.9%)	2286 (21.0%)	5078 (12.9%)	131 (14.5%)	< 0.001
Transfusion	35,003 (40.5%)	7061 (40.1%)	8460 (47.9%)	5216 (48.0%)	13,866 (35.2%)	400 (44.3%)	< 0.001
Valve surgery—mechanical prothesis	3346 (3.9%)	992 (5.6%)	733 (4.2%)	273 (2.5%)	1304 (3.3%)	44 (4.9%)	< 0.001
Valve surgery—biological prothesis	13,594 (15.7%)	3097 (17.6%)	3292 (18.6%)	1821 (16.8%)	5221 (13.3%)	163 (18.1%)	< 0.001
Outcomes and discharge/referral pathways							
In-hospital mortality	12,852 (14.9%)	1618 (9.2%)	3271 (18.5%)	2036 (18.7%)	5817 (14.8%)	110 (12.2%)	< 0.001
Discharge to another hospital	28,801 (33.3%)	6099 (34.6%)	6247 (35.4%)	3112 (28.6%)	13,051 (33.1%)	292 (32.3%)	< 0.001
Discharge to rehabilitation	8436 (9.8%)	2026 (11.5%)	1663 (9.4%)	986 (9.1%)	3674 (9.3%)	87 (9.6%)	< 0.001
Discharge to nursing home	1267 (1.5%)	213 (1.2%)	243 (1.4%)	287 (2.6%)	506 (1.3%)	18 (2.0%)	< 0.001
Discharge others	35,111 (40.6%)	7662 (43.5%)	6249 (35.4%)	4445 (40.9%)	16,359 (41.5%)	396 (43.9%)	< 0.001

*BCNIE* blood culture-negative infective endocarditis, *COPD* chronic obstructive pulmonary disease, *PAH* pulmonary arterial hypertension

### Incidence

**Fig. 2:**
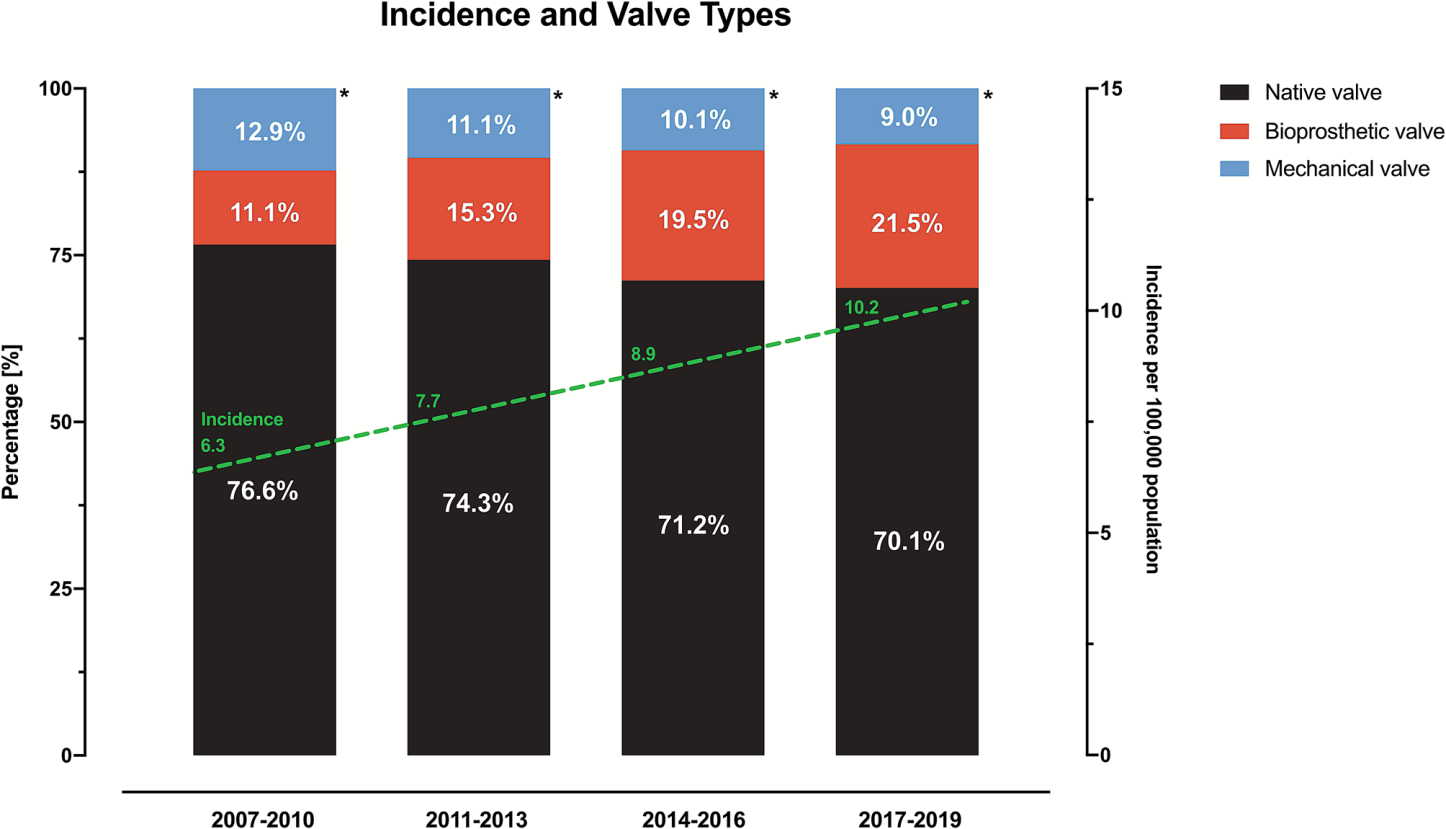
Temporal increase in IE incidence. Shown is the incidence of IE per 100,000 population in year groups (dashed line in green). Colors in the stacked columns indicate valve or device types. * = derivation from 100% is due to patients with multiple valve implantations at index hospitalization

Between 2007 and 2019, the annual mean incidence of IE increased from 6.3 per 100,000 population (20,716 cases) in 2007–2010 to 10.2 per 100,000 population (25,346 cases) in 2017–2019 (Graphical abstract, Fig. 2).

### Patient characteristics

Patients with IE often had CV comorbidities including hypertension (*n* = 38,792; 44.9%), atrial fibrillation (*n* = 32,955; 38.1%), heart failure (HF) (*n* = 34,233; 39.6%), chronic kidney disease (CKD) (*n* = 29,116; 33.7%), diabetes (*n* = 23,556; 27.2%), and chronic obstructive pulmonary disease (COPD) (*n* = 7576; 8.8%). Approximately one in five patients with IE presented with acute kidney failure (17,700; 20.5%), 17,463 (20.2%) patients had sepsis, and 3,927 (4.5%) patients had septic shock.

Most patients with IE presented with native valve (*n* = 62,977; 72.8%) followed by 17.2% (*n* = 14,839) of IE patients with bioprosthetic prothesis and 10.7% (n = 9,226) of IE patients with mechanical prothesis (Graphical abstract, Fig. 2, Table 1).

**Fig. 3:**
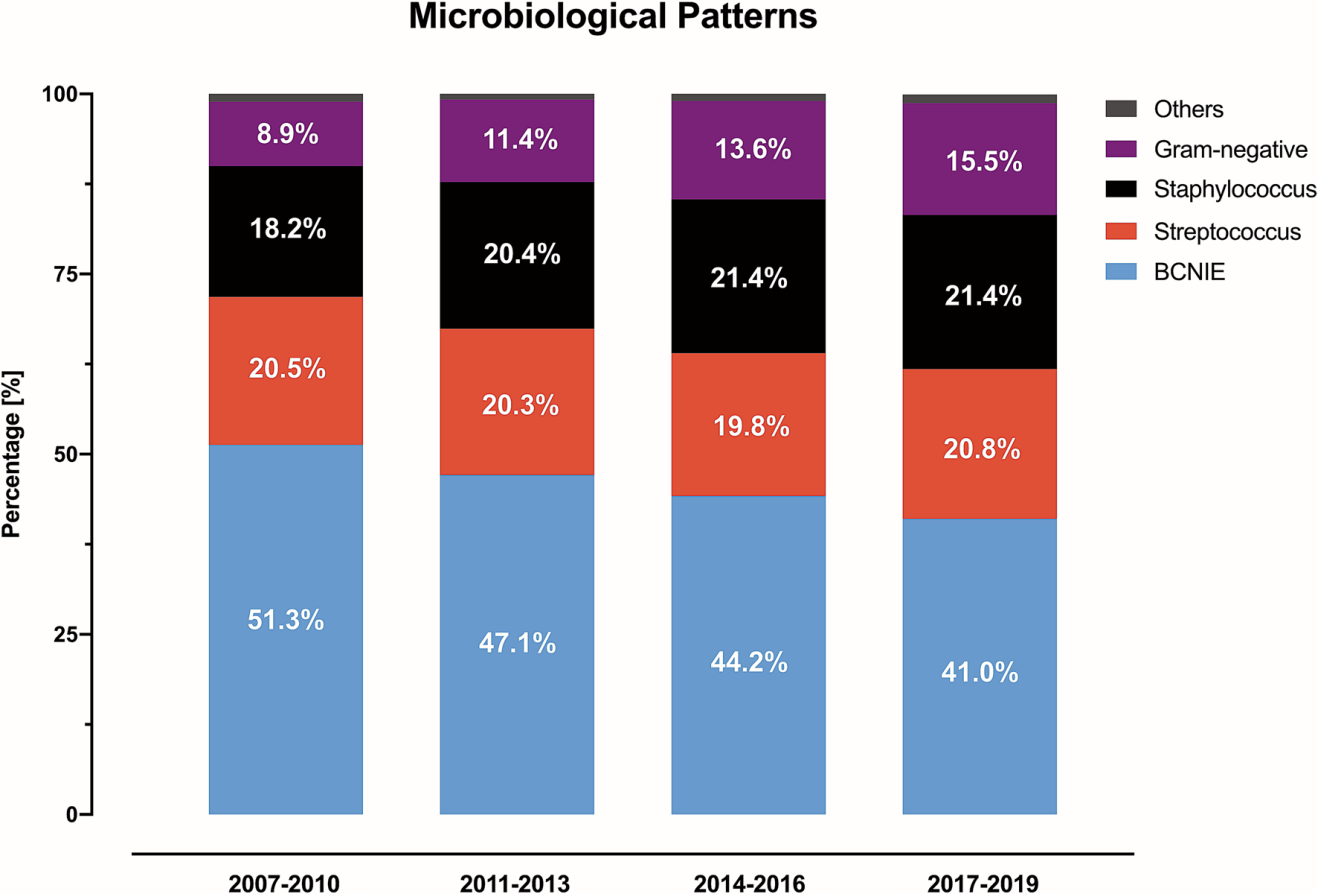
Temporal trends in bacterial pathogens causing IE over time. *BCNIE* blood culture-negative infective endocarditis

*Staphylococcus* (*n* = 17,673; 20.4%), s*treptococcus* (*n* = 17,618; 20.4%), and Gram-negative (*n* = 10,866; 12.6%) were the most common pathogens identified (Fig. 3). BCNIE was observed in 45.6% (*n* = 39,409) of the cases (Table 1). Patients with IE and Gram-negative bacteremia were older and had more CV comorbidities (hypertension, atrial fibrillation, HF, CKD, diabetes, COPD) compared to patients with IE and other microbiological patterns.

The majority of patients (*n* = 63,238, 73.1%) underwent transoesophageal echocardiography (TOE) for diagnosis of IE with a trend towards increased use of this diagnostic tool over time (66.3% in 2007–2010 to 76.5% in 2017–2019) (Table 1). During hospitalization for IE, 13,594 (15.7%) patients received bioprosthetic valve replacement, and 3,346 (3.9%) patients underwent mechanical valve replacement. In addition, 3,388 (3.9%) patients with IE underwent extraction of an implanted cardiac electronic device (pacemaker or defibrillator) during the hospitalization for IE.

### Temporal trends in patient characteristics, microbiological patterns and valve types

The mean age and the proportion of IE patients with hypertension, atrial fibrillation, HF, CKD, diabetes, and COPD increased significantly over time (Supplementary Tables S2–6).

IE cases with *staphylococcus* (18.2% in 2007–2010 to 21.4% in 2017–2019) and Gram-negative bacteremia (8.9% in 2007–2010 to 15.5% in 2017–2019) were increasing over time, whereas the proportion of BCNIE was gradually decreasing (51.3% in 2007–2010 to 41.0% in 2017–2019). Bacteremia with *streptococcus* remained relatively stable over time.

The proportion of IE patients with mechanical and native valves decreased in the past decade, whereas the proportion of IE patients with bioprosthetic valves continuously increased (Fig. 2, Supplementary Table S2).

### In-hospital mortality and discharge/referral pathways

The overall in-hospital mortality was 14.9% with a trend towards increasing in-hospital mortality rates over time (13.4% in 2007–2010 to 16.5% in 2017–2019) (Table 1, Supplementary Fig. S1, Supplementary Table S2).

In-hospital mortality rates stratified by pathogen organism confirmed increasing rates over time for all pathogens examined (*staphylococcus*: 16.1% in 2007–2010 to 20.2% in 2017–2019; *streptocococcus*: 8.8% in 2007–2010 to 10.5% in 2017–2019; Gram-negative: 15.5% in 2007–2010 to 20.8% in 2017–2019; BCNIE: 14.1% in 2007–2010 to 16.0% in 2017–2019, respectively) (Supplementary Fig. S1, Supplementary Tables S3–6).

Mean duration of index hospitalization was 24 (± 18) days, the majority of patients hospitalized due to IE were discharged to another hospital 33.3% (*n* = 28,801), whereas 9.8% (*n* = 8436) of patients were discharged to rehabilitation and 1.5% (*n* = 1267) of patients were discharged to nursing home (Table 1). Detailed information on discharge/referral pathways are shown in Supplementary Tables S2–6.

### Independent predictors of in-hospital mortality

**Fig. 4:**
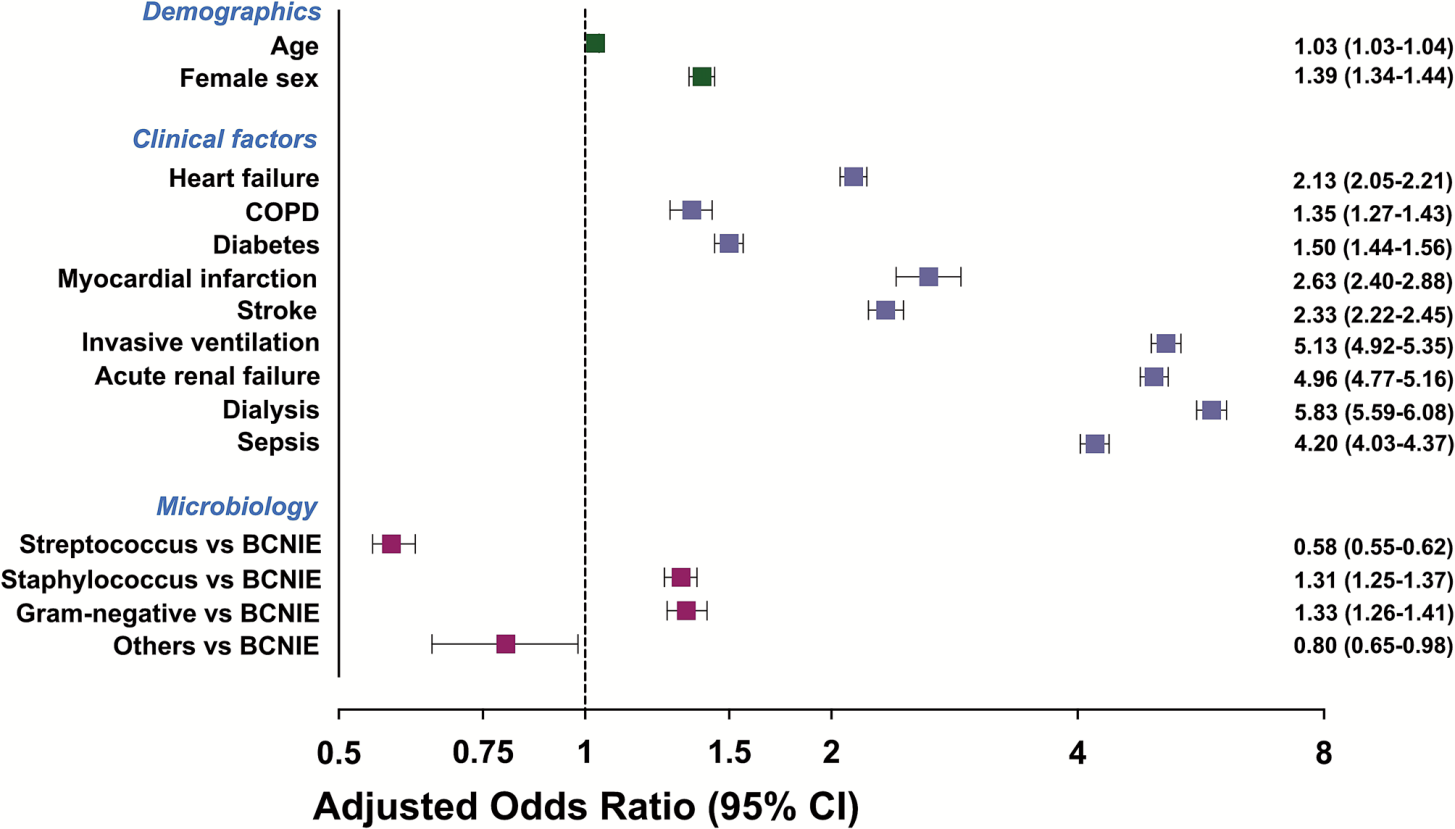
Factors associated with in-hospital mortality in patients hospitalized with endocarditis. *BCNIE* blood culture-negative infective endocarditis, *CI* confidence interval, *COPD* chronic obstructive pulmonary disease

The following clinical characteristics were associated with higher in-hospital mortality: advanved age (odds ratio [OR] 1.03, 95%-confidence interval [CI] 1.03–1.04; *p* < 0.001), female sex (OR = 1.39, 95%-CI 1.34–1.44; *p* < 0.001), HF (OR = 2.13, 95%-CI 2.05–2.21; *p* < 0.001), COPD (OR = 1.35, 95%-CI 1.27–1.43; *p* < 0.001), diabetes (OR = 1.50, 95%-CI 1.44–1.56; *p* < 0.001), myocardial infarction (OR = 2.63, 95%-CI 2.40–2.88; *p* < 0.001), stroke (OR = 2.33, 95%-CI 2.22–2.45; *p* < 0.001), need for invasive ventilation (OR = 5.13, 95%-CI 4.92–5.35; *p* < 0.001), acute renal failure (OR = 4.96, 95%-CI 4.77–5.16; *p* < 0.001), need for dialysis (OR = 5.83, 95%-CI 5.59–6.08; *p* < 0.001), and sepsis (OR = 4.20, 95%-CI 4.03–4.37; *p* < 0.001) (Graphical abstract, Fig. 4).

Compared to IE patients with BCNIE, patients with *staphylococcus* and Gram-negative pathogens detected in blood cultures had a higher in-hospital mortality compared to BCNIE (*staphylococcus* OR = 1.31, 95%-CI 1.25–1.37; *p* < 0.001; Gram-negative pathogens OR = 1.33, 95%-CI 1.26–1.41; *p* < 0.001), whereas *streptococcus* and others had a lower risk of in-hospital mortality (*streptococcus* OR = 0.58, 95%-CI 0.55–0.63; *p* < 0.001; others OR = 0.80, 95%-CI 0.65–0.98; *p* = 0.03) (Graphical abstract, Fig. 4).

## Discussion

In this nationwide cohort including almost 90,000 IE cases, we found that (1) the incidence of IE increased by 61.9% in the last 12 years; (2) IE remains associated with high in-hospital morbidity and mortality; (3) age, the number of CV comorbidities, and implanted heart valves or cardiac devices increase over time in patients hospitalized with IE; 4) pathogens changed over time: *staphylococcus* and *streptococcus* were the predominant causative microorganisms; *staphylococcus* and Gram-negative bacteremia increased; and 5) in-hospital mortality during hospitalization was associated with advanced age, stroke, HF, diabetes, COPD, myocardial infarction, acute renal failure, the need for invasive ventilation or dialysis, sepsis, and infection with *staphylococcus* and Gram-negative bacteria.

### Incidence

IE is still considered to be an infrequent disease with incidence rates reported ranging from 3 to 9 per 100,000 population [7, 12, 13]. In this cohort, a gradually increase in incidence was documented from 6.3 to 10.2 per 100,00 population between 2007 and 2019 matching recent data reported from the US [14]. Notably, this increase seems primarily attributable to health care-associated or nosocomial infections and growing patient cohorts at increased risk of IE, including those with advanced age, congenital heart disease, requiring hemodialysis, implantable pacing devices or prosthetic heart valves [1, 15, 16]. For the latter, the combined number of open-heart and transcatheter aortic valve replacements (TAVR) in Germany has increased by more than 70% during the investigation period (*n* = 21,312 in 2007 vs. *n* = 36,650 in 2019), especially due to an increased number of TAVRs performed (*n* = 157 in 2007 vs. *n* = 15,304 in 2019) [17].

### Trends in patient characteristics, microbiological patterns and valve types

A large and growing proportion of affected patients with IE is characterized by an advanced age profile, numerous CV comorbidities, and presence of prosthetic heart valves or other intra-cardiac devices [15]. Consistent with previous literature, we observed an advanced age profile and a high burden of CV comorbidities in patients hospitalized with IE [1]. Our study demonstrated that key patient characteristics have changed substantially over time. In particular, patient age and the burden of CV comorbidities increased considerably during the study period (2007–2010 vs. 2017–2019 for all following: mean age in years: 65.1 vs. 67.1; atrial fibrillation: 32.0% vs. 42.7%; HF 33.0% vs. 45.2%; hypercholesterinemia 16.6% vs. 26.9%, respectively). These changes in demographic and clinical characteristics are in line with recent reports highlighting the overall vulnerable and elderly patient population [15, 18]. In our analysis, ~ 3/4 of the patients with IE underwent TOE reflecting the importance of this diagnostic tool and the current guideline recommendations for diagnosis of IE [19, 20].

IE is a heterogeneous disease with outcomes depending on the underlying pathogen identified as well as on the presence of prosthetic material [5]. A substantial shift in IE epidemiology has been identified favoring *staphylococcus aureus* as the most common causative pathogen in the Western world [7]. Although both *streptococcus* and *enterococcus* species are considered together in our study, IE due to *staphylococcus* species outnumbered all other forms of IE with a pathogen identified between 2017 and 2019. In our analysis, the overall temporal trends in microbiological patterns with decreasing proportions for BCNIE and IE with *streptococcus* species in favor of IE due to *staphylococcus* species are confirmed [14].

Regarding Gram-negative pathogens, our analysis combines various species that have traditionally been posing a challenge to grow in culture [21]. HACEK species have been reported as accountable for 5–10% of IE cases and non-HACEK for about 2% [5]. Although information on the individual proportion of HACEK vs. non-HACEK cases is missing in this analysis, the noted increase of almost 75% from the 2007–2010 to the 2017–2019 period as well as the association with a higher risk of in-hospital mortality (OR = 1.3 for in-hospital mortality compared to BCNIE) underlines the necessity to increase preventive actions and treatment strategies for this subset of IE patients.

In our study, we found that the proportion of prosthetic valves affected by IE substantially increased, accentuated for cases with *staphylococcus* (+ 61.6%) and Gram-negative pathogens (+ 59.1%). Notably, *staphylococcus* is associated with adverse outcomes in this analysis (OR = 1.31 for in-hospital mortality as compared to BCNIE), due to its antibiotic resistance and frequent involvement in prosthetic valve IE [14, 22].

Whereas the number of mechanical valve replacements decreased over time, a growing number of patients received biological valve replacement during index hospitalization (+ 41.4% increase 2007–2010 vs. 2017–2019: 12.5% vs. 17.7%). Due to the data available, it cannot be differentiated whether the individual patient primarily received valve surgery and suffered from IE in the post-operative course or if the patient initially was hospitalized for IE and then operated on due to IE complications. It can be speculated that the latter was more frequent in those patients [7]. Additionally, the rate of lead extraction/device exchange of 25.9% was lower than in the current literature and could be explained by the fact that in certain cases the implanted cardiac devices were not considered causative for IE or patients with IE were transferred to specialized hospitals for lead extraction/device exchange [23, 24].

### Outcomes and discharge/referral pathways

The in-hospital mortality is estimated ~ 15% and 6-month mortality rates are estimated ~ 22% in patients with IE [25, 26]. Among 1-year survivors, reported long-term mortality rates were 3% at 2 years, 10% at 5 years, 16% at 10 years, 25% at 15%, and 29% at 20 years, respectively [3]. Consistently, we observed high in-hospital mortality rates (~ 15%) in patients hospitalized due to IE. In our study, slightly increasing in-hospital mortality rates over the study interval could be explained in part by an overall elderly patient population with higher incidence of comorbid diseases in recent years (in-hospital mortality: 13.4% in 2007–2010 to 16.5% in 2017–2019).

Another important clinical observation in our study was that IE cases with *staphylococcus* and Gram-negative pathogens were associated with a higher risk of in-hospital mortality compared to other forms of IE. This finding is consistent with recent reports and highlights the markely increased risk of adverse outcomes and complications, especially seen in IE patients with *staphylococcus* and Gram-negative pathogens [27]. This is of particular importance since incidence rates of *staphylococcus* and Gram-negative pathogens are increasing and *staphylococcus aureus* is now the leading cause of IE in many regions of the world [11].

Although the finding that typical complications of IE are associated with adverse outcomes itself is not surprising, the extent of impact as implied by this analysis seems worth to consider. IE complications can be divided into three principal groups: first, cardiac complications including worsening HF or myocardial infarction. Second, worsening of non-cardiac organ function and comorbidities comprising diabetes, COPD, stroke, and acute kidney failure. Third, factors associated with the need for intensive care treatment including invasive ventilation, dialysis, and sepsis. Out of these, especially the latter is associated with excess mortality rates as expressed by ORs ranging from 4.2 to 5.8 for in-hospital mortality.

Several studies indicate that patients with IE experience deconditioning and reduced quality of life post-discharge [28]. In our analysis, a relevant proportion of patients was discharged to another hospital (~ 35%) and ~ 10% of patients with IE were discharged primarily to rehabilitation after index hospitalization. These findings support the overall high need for comprehensive rehabilitation in this critically ill patient population.

### Outlook and perspectives

Considering rising incidence rates, trends in microbiological patterns and increasing rates of in-hospital mortality, there is need to further develop new preventive and therapeutic approaches. This appears to be particularly important in the context of the also markedly increasing implantation rates of prosthetic heart valves, particularly covering the area of transcatheter aortic valve replacement. As this analysis itself is limited to Germany, the number of implanted heart valves is increasing in all high-income countries [29]. It has to be further investigated to what extent the rise in incidence of IE is attributable to the increasing use of transcatheter heart valve procedures and intra-cardiac devices; or major demographic changes such as an overall aging general population with an increasing burden of CV comorbidities led to the increase in incidence of IE over time. Aside from an overall vigilance and meticulously follow-up strategies, patients diagnosed with IE should be referred to tertiary care or specialized IE centers, according to the current version of the guidelines [19]. While the restriction of antibiotic prophylaxis recommended by the American and European guidelines has not yielded into an increase in IE due to *streptococcus* species, its preventive potential in the setting of non-*streptococcus* bacteremia as well as in patients with prosthetic heart valves has to be monitored and studied in more detail [30].

### Limitations

Our study has several limitations that should be acknowledged. As only in-hospital outcome is available, information on long-term complications and mortality rates after discharge from index hospitalization are missing. Additionally, only the diagnosis of IE, but not the valve(s) affected were available. Only coded diagnoses and procedures are available, preventing the exact temporal reconstruction of the individual hospital course. Especially regarding the antibiotic prophylaxis and causal pathogens, a more detailed listing would be of paramount interest, regarding the proportion of *staphylococcus aureus* or *enterococcus* bacteremia. Due to the nature of the dataset derived from ICD-10-GM code or OPS codes, further discrimination between staphylococcus aureus and the streptococcus group is not available. This represents a significant limitation and has to be considered when interpreting our results. Differentiation between community-acquired and nosocomial IE was not available using this dataset. Finally, as our dataset is derived from German hospitals and based on administrative data only, generalizability to other healthcare systems might be limited and conclusions must be drawn with utmost caution.

## Conclusions

In this nationwide cohort of patients with IE, patient characteristics were trending towards an advanced age profile and an increased burden of CV comorbidities, while the incidence increased between 2007 and 2019 with an overall high in-hospital mortality (~ 15%)*.* The predominant causative microorganisms were *staphylococcus* and *streptococcus*. While cases with *staphylococcus* and Gram-negative IE were considerably increasing over time, the proportion BCNIE was decreasing. Both, IE cases with *staphylococcus* bacteremia and Gram-negative IE were associated with higher risk of in-hospital mortality compared to other subforms. Our results highlight the need for new preventive strategies and interventions with carefully selected and differentiated antibiotic therapy in patients hospitalized with IE.

### Supplementary Information

Below is the link to the electronic supplementary material.

Supplementary file1 (DOCX 82 KB)

## Data Availability

The data underlying this article will be shared on reasonable request to the corresponding author.

## Abbreviations

CKD: Chronic kidney disease
CI: Confidence interval
COPD: Chronic obstructive pulmonary disease
CV: Cardiovascular
HACEK: Haemophilus species, aggregate bacter actinomycetemcomitans, cardiobacterium hominis, eikenella corrodens, kingella species
HF: Heart failure
ICD-10-GM: German modification of the International Statistical Classification of Diseases and Related Health Problems, 10th revision
IE: Infective endocarditis
OPS: “*Operationen und Prozedurenschlüssel”*; *German;* Operational and procedural codes adapted from the International Classification of Procedures in Medicine issued by the World Health Organization
OR: Odds ratio
SD: Standard deviation
TAVR: Transcatheter aortic valve replacement
TOE: Transoesophageal echocardiography
US: United States
