# Supplementary material for: Temporal trends in incidence, patient characteristics, microbiology and in-hospital mortality in patients with infective endocarditis: a contemporary analysis of 86,469 cases between 2007 and 2019
Source: Clin Res Cardiol. 2022 Sep 12;113(2):205–15. doi: 10.1007/s00392-022-02100-4 (PMC10850016; doi:10.1007/s00392-022-02100-4)
Supplement: Supplementary file 1 — Supplementary file1 (DOCX 82 KB) [file 392_2022_2100_MOESM1_ESM.docx]

**Supplementary Material**

Supplementary Material to: Becher et al., Temporal Trends in Incidence, Patient Characteristics, Microbiology and In-Hospital Mortality in Patients with Infective Endocarditis: A Contemporary Analysis of 86,469 Cases between 2007 and 2019

**Table of Contents**   **Page number**

**Supplementary Tables**

**Supplementary Table S1.** 2

Variable definitions

**Supplementary Table S2.** 3

Baseline characteristics in patients with infective endocarditis and stratified

by year groups

**Supplementary Table S3.** 6

Baseline characteristics in patients with infective endocarditis and stratified

by microbiology between 2007 and 2010

**Supplementary Table S4.** 9

Baseline characteristics in patients with infective endocarditis and stratified

by microbiology between 2011 and 2013

**Supplementary Table S5.** 12

Baseline characteristics in patients with infective endocarditis and stratified

by microbiology between 2014 and 2016

**Supplementary Table S6.** 15

Baseline characteristics in patients with infective endocarditis and stratified

by microbiology between 2017 and 2019

**Supplementary Table S1.** Variable definitions

| **Variable** | **Definition** |
| --- | --- |
| Hypertension | ICD-10-GM: I10 |
| Hypercholesterinemia | ICD-10-GM: E78 |
| Diabetes | ICD-10-GM: E10-E11 |
| Peripheral vascular disease | ICD-10-GM: I70.2 |
| Streptococcus positive BC | ICD-10-GM: B95.0-95.5 |
| Staphylococcus positive BC | ICD-10-GM: B95.6-95.8 |
| Gram-negative positive BC | ICD-10-GM: B96.2, B96.3, B96.5, B96.6 |
| Others (positive BC) | ICD-10-GM: B96.0, B96.7, B96.8 |
| Prior stroke | ICD-10-GM: I63-64 |
| Ischemic stroke | ICD-10-GM: I63, I65, I66 |
| Hemorrhagic stroke | ICD-10-GM: I60-62 |
| Atrial fibrillation | ICD-10-GM: I48 |
| Chronic obstructive pulmonary disease | ICD-10-GM: J44 |
| Hypercholesterinemia | ICD-10-GM: E78 |
| Peripheral vascular disease | ICD-10-GM: I70.2 |
| Pulmonary hypertension | ICD-10-GM: I27.0, I27.2, I27.20 |
| Immunosuppression | ICD-10-GM: D80-89 |
| Acute renal failure | ICD-10-GM: N17 |
| Chronic kidney disease | ICD-10-GM: N18-19 |
| Malignancy | ICD-10-GM: C00-97 |
| History of coronary bypass graft | ICD-10-GM: Z95.1 |
| Acute myocardial infarction | ICD-10-GM: I21 |
| Severe pulmonary embolism | ICD-10-GM: I26.0 |
| Acute myocarditis | ICD-10-GM: I40-41 |
| Sepsis | ICD-10-GM: A41 |
| Septic shock | ICD-10-GM: R57.2 |
| Implanted pacing device | ICD-10-GM: Z95.0 |
| Coronary bypass graft | OPS: 5-361, 5-362 |
| Invasive ventilation | OPS: 8-701 |
| Non-invasive ventilation | OPS: 8-706 |
| Change of pacing device | OPS: 5-378 |
| Dialysis | OPS: 8-853, 8-854, 8-855 |
| Transfusion | OPS: 8-800 |
| Valve surgery - biological prothesis | OPS: 5-351.01-07, 5-351.11-13, 5-351.21-23,  5-351.31-33, 5-351.37, 5-351.41-43, 5-351.0x, 5-351.1x, 5-351.2x, 5-351.3x, 5-351.4x |
| Valve surgery - mechanical prothesis | OPS: 5-351.04, 5-351.14, 5-351.24, 5-351.34, 5-351.44 |

Abbreviations: BC, Blood culture; ICD-10-GM, German modification of the International Statistical Classification of Diseases and Related Health Problems, 10th revision; OPS, German Operational and Procedural codes.

**Supplementary Table S2.** Baseline characteristics in patients with infective endocarditis and stratified by year groups

| **Variables** | **Overall cohort** | **2007-2010** | **2011-2013** | **2014-2016** | **2017-2019** | **p-value** |
| --- | --- | --- | --- | --- | --- | --- |
|  | **(n=86469)** | **(n=20716)** | **(n=18446)** | **(n=21961)** | **(n=25346)** |  |
| ***Demographics*** | | | | | | |
| Age (years) | 66.5 ± 14.7 | 65.1 ± 14.8 | 66.2 ± 14.8 | 66.9 ± 14.7 | 67.6 ± 14.5 | <0.001 |
| Sex, female | 27534 (31.8%) | 6907 (33.3%) | 6057 (32.8%) | 6986 (31.8%) | 7584 (29.9%) | <0.001 |
| Length of hospital stay | 23.9 ± 18.3 | 24.4 ± 18.7 | 23.5 ± 17.9 | 24.2 ± 18.7 | 23.6 ± 17.8 | <0.001 |
| ***Comorbidities*** | | | | | | |
| Hypertension | 38792 (44.9%) | 8216 (39.7%) | 8209 (44.5%) | 10117 (46.1%) | 12250 (48.3%) | <0.001 |
| Diabetes | 23556 (27.2%) | 5226 (25.2%) | 4981 (27.0%) | 6188 (28.2%) | 7161 (28.6%) | <0.001 |
| COPD | 7576 (8.8%) | 1638 (7.9%) | 1555 (8.4%) | 2041 (9.3%) | 2342 (9.2%) | <0.001 |
| PAH | 6709 (7.8%) | 1200 (5.8%) | 1392 (7.6%) | 1988 (9.1%) | 2129 (8.4%) | <0.001 |
| History of Stroke | 7137 (8.3%) | 1531 (7.4%) | 1422 (7.7%) | 1886 (8.6%) | 2298 (9.1%) | <0.001 |
| Atrial fibrillation | 32955 (38.1%) | 6625 (32.0%) | 6669 (36.2%) | 8828 (40.2%) | 10833 (42.7%) | <0.001 |
| Hypercholesterinemia | 19319 (22.3%) | 3438 (16.6%) | 3850 (20.9%) | 5217 (23.8%) | 6814 (26.9%) | <0.001 |
| Peripheral vascular disease | 5126 (5.9%) | 978 (4.7%) | 1051 (5.7%) | 1389 (6.3%) | 1708 (6.7%) | <0.001 |
| Chronic kidney disease | 29116 (33.7%) | 6466 (31.2%) | 6378 (34.6%) | 7636 (34.8%) | 8636 (34.1%) | <0.001 |
| Heart failure | 34233 (39.6%) | 6825 (33.0%) | 6810 (36.9%) | 9154 (41.7%) | 11444 (45.2%) | <0.001 |
| History of bypass surgery | 7130 (8.3%) | 1513 (7.3%) | 1513 (8.2%) | 1961 (8.9%) | 2143 (8.5%) | <0.001 |
| Cardiac valve status |  |  |  |  |  |  |
| Mechanical prothesis | 9226 (10.7%) | 2667 (12.9%) | 2047 (11.1%) | 2215 (10.1%) | 2297 (9.1%) | <0.001 |
| Biological prothesis | 14839 (17.2%) | 2295 (11.1%) | 2820 (15.3%) | 4275 (19.5%) | 5449 (21.5%) | <0.001 |
| Native valve | 62977 (72.8%) | 15869 (76.6%) | 13707 (74.3%) | 15625 (71.2%) | 17776 (70.1%) | <0.001 |
| Implanted pacing device | 13041 (15.1%) | 2411 (11.6%) | 2615 (14.2%) | 3696 (16.8%) | 4319 (17.04%) | <0.001 |
| Change or extraction of pacing device | 3388 (25.9%) | 584 (24.2%) | 638 (24.3%) | 1056 (28.5%) | 1110 (25.7%) | <0.001 |
| Malignancy | 4289 (4.0%) | 1010 (4.9%) | 897 (4.9%) | 1120 (5.1%) | 1262 (5.0%) | 0.656 |
| Immunosuppression | 303 (0.4%) | 81 (0.4%) | 61 (0.3%) | 83 (0.4%) | 78 (0.3%) | 0.395 |
| ***Clinical presentation*** | | | | | | |
| Myocardial infarction | 2312 (2.7%) | 498 (2.4%) | 507 (2.8%) | 608 (2.8%) | 699 (2.8%) | 0.054 |
| Myocarditis | 115 (0.1%) | 30 (0.1%) | 20 (0.1%) | 31 (0.1%) | 34 (0.1%) | 0.76 |
| Severe pulmonary embolism | 249 (0.3%) | 45 (0.2%) | 48 (0.3%) | 71 (0.3%) | 85 (0.3%) | 0.071 |
| Acute renal failure | 17700 (20.5%) | 2516 (12.2%) | 2777 (15.1%) | 5008 (22.8%) | 7399 (29.2%) | <0.001 |
| Stroke | 9810 (11.4%) | 2059 (9.9%) | 1999 (10.8%) | 2589 (11.8%) | 3163 (12.5%) | <0.001 |
| Ischemic | 8713 (10.1%) | 1803 (8.7%) | 1752 (9.5%) | 2312 (10.5%) | 2846 (11.2%) | <0.001 |
| Hemorrhagic | 1687 (2.0%) | 358 (1.7%) | 351 (1.9%) | 445 (2.0%) | 533 (2.1%) | 0.025 |
| Sepsis | 17463 (20.2%) | 3631 (17.5%) | 3578 (19.4%) | 4759 (21.7%) | 5495 (21.7%) | <0.001 |
| Septic shock | 3927 (4.5%) | 174 (0.8%) | 836 (4.5%) | 1265 (5.8%) | 1652 (6.5%) | <0.001 |
| ***Microbiology*** | | | | | | |
| Streptococcus | 17618 (20.4%) | 4238 (20.5%) | 3738 (20.3%) | 4358 (19.8%) | 5284 (20.8%) | <0.001 |
| Staphylococcus | 17673 (20.4%) | 3770 (18.2%) | 3772 (20.4%) | 4695 (21.4%) | 5436 (21.4%) | <0.001 |
| Gram-negative | 10866 (12.6%) | 1849 (8.9%) | 2102 (11.4%) | 2992 (13.6%) | 3923 (15.5%) | <0.001 |
| BCNIE | 39409 (45.6%) | 10625 (51.2%) | 8690 (47.1%) | 9704 (44.2%) | 10390 (41.0%) | <0.001 |
| Others | 903 (1.0%) | 234 (1.1%) | 144 (0.8%) | 212 (1.0%) | 313 (1.2%) | <0.001 |
| ***Treatments*** | | | | | | |
| Transoesophageal echocardiography | 63238 (73.1%) | 13743 (66.3%) | 13402 (72.6%) | 16681 (75.9%) | 19412 (76.5%) | <0.001 |
| Invasive ventilation | 14232 (16.5%) | 3090 (14.9%) | 2969 (16.1%) | 3844 (17.5%) | 4329 (17.1%) | <0.001 |
| Non-invasive ventilation | 5664 (6.6%) | 827 (4.0%) | 1086 (5.9%) | 1700 (7.7%) | 2051 (8.1%) | <0.001 |
| Dialysis | 12827 (14.8%) | 2957 (14.3%) | 2803 (15.2%) | 3284 (15.0%) | 3783 (14.9%) | 0.058 |
| Transfusion | 35003 (40.5%) | 8202 (39.6%) | 7781 (42.2%) | 8921 (40.6%) | 10099 (39.8%) | <0.001 |
| Valve surgery - mechanical prothesis | 3346 (3.9%) | 1186 (5.7%) | 759 (4.1%) | 760 (3.5%) | 641 (2.5%) | <0.001 |
| Valve surgery - biological prothesis | 13594 (15.7%) | 2587 (12.5%) | 2898 (15.7%) | 3633 (16.5%) | 4476 (17.7%) | <0.001 |
| ***Outcomes and discharge/referral structure*** | | | | | | |
| In-hospital mortality | 12852 (14.9%) | 2784 (13.4%) | 2629 (14.3%) | 3253 (14.8%) | 4186 (16.5%) | <0.001 |
| Discharge to another hospital | 28801 (33.3%) | 6358 (30.7%) | 6073 (32.9%) | 7470 (34.0%) | 8900 (35.1%) | <0.001 |
| Discharge to rehabilitation | 8436 (9.8%) | 2255 (10.9%) | 1940 (10.5%) | 2148 (9.8%) | 2093 (8.3%) | <0.001 |
| Discharge to nursing home | 1267 (1.5%) | 271 (1.3%) | 263 (1.4%) | 333 (1.5%) | 400 (1.6%) | 0.095 |
| Discharge others | 35111 (40.6%) | 9046 (43.7%) | 7541 (40.9%) | 8757 (39.9%) | 9767 (38.5%) | <0.001 |

Abbreviations: BCNIE, blood culture-negative infective endocarditis; COPD, chronic obstructive pulmonary disease; PAH, pulmonary arterial hypertension.

**Supplementary Table S3.** Baseline characteristics in patients with infective endocarditis and stratified by microbiology between 2007 and 2010

| **Variables** | **2007-2010** | **Streptococcus** | **Staphylococcus** | **Gram-negative** | **BCNIE** | **Others** | **p-value** |
| --- | --- | --- | --- | --- | --- | --- | --- |
|  | **(n=20716)** | **(n=4238)** | **(n=3770)** | **(n=1849)** | **(n=10625)** | **(n=234)** |  |
| ***Demographics*** | | | | | | | |
| Age (years) | 65.1 ± 14.8 | 64.5 ± 14.8 | 64.4 ± 15.5 | 69.5 ± 12.7 | 64.9 ± 14.8 | 66.2 ± 14.5 | <0.001 |
| Sex, female | 6907 (33.3%) | 1121 (26.5%) | 1195 (31.7%) | 826 (44.7%) | 3675 (34.6%) | 90 (38.7%) | <0.001 |
| Length of hospital stay | 24.4 ± 18.7 | 25.8 ± 17.1 | 25.5 ± 18.9 | 34.2 ± 24.3 | 21.7 ± 17.4 | 30.0 ± 21.9 | <0.001 |
| ***Comorbidities*** | | | | | | | |
| Hypertension | 8216 (39.7%) | 717 (16.9%) | 640 (17.0%) | 376 (20.3%) | 1664 (15.7%) | 41 (17.5%) | <0.001 |
| Diabetes | 5226 (25.2%) | 998 (23.6%) | 1072 (28.4%) | 643 (34.8%) | 2433 (22.9%) | 80 (34.2%) | <0.001 |
| COPD | 1638 (7.9%) | 321 (7.6%) | 320 (8.5%) | 176 (9.5%) | 807 (7.6%) | 14 (6.0%) | 0.021 |
| PAH | 1200 (5.8%) | 266 (6.3%) | 240 (6.4%) | 135 (7.3%) | 549 (5.2%) | 10 (4.3%) | <0.001 |
| History of Stroke | 1531 (7.4%) | 262 (6.2%) | 366 (9.7%) | 168 (9.1%) | 720 (6.8%) | 15 (6.4%) | <0.001 |
| Atrial fibrillation | 6625 (32.0%) | 1338 (31.6%) | 1358 (36.0%) | 694 (37.5%) | 3165 (29.8%) | 70 (29.9%) | <0.001 |
| Hypercholesterinemia | 3438 (16.6%) | 717 (16.9%) | 640 (17.0%) | 376 (20.3%) | 1664 (15.7%) | 41 (17.5%) | <0.001 |
| Peripheral vascular disease | 978 (4.7%) | 125 (3.0%) | 264 (7.0%) | 131 (7.1%) | 444 (4.2%) | 14 (6.0%) | <0.001 |
| Chronic kidney disease | 6466 (31.2%) | 1184 (27.9%) | 1350 (35.8%) | 765 (41.4%) | 3093 (29.1%) | 74 (31.6%) | <0.001 |
| Heart failure | 6825 (33.0%) | 1336 (31.5%) | 1331 (35.3%) | 694 (37.5%) | 3392 (31.9%) | 72 (30.8%) | <0.001 |
| History of bypass surgery | 1513 (7.3%) | 292 (6.9%) | 307 (8.1%) | 143 (7.7%) | 756 (7.1%) | 15 (6.4%) | 0.176 |
| Cardiac valve status |  |  |  |  |  |  |  |
| Mechanical prothesis | 2667 (12.9%) | 577 (13.6%) | 464 (12.3%) | 218 (11.8%) | 1383 (13.0%) | 25 (10.7%) | 0.179 |
| Biological prothesis | 2295 (11.1%) | 561 (13.2%) | 412 (10.9%) | 239 (12.9%) | 1053 (9.9%) | 30 (12.8%) | <0.001 |
| Native valve | 15869 (76.6%) | 3132 (73.9%) | 2914 (77.3%) | 1401 (75.8%) | 8241 (77.6%) | 181 (77.4%) | <0.001 |
| Implanted pacing device | 2411 (11.6%) | 412 (9.7%) | 603 (16.0%) | 228 (12.3%) | 1148 (10.8%) | 20 (8.6%) | <0.001 |
| Change or extraction of pacing device | 584 (24.2%) | 64 (15.5%) | 220 (36.4%) | 48 (21.0%) | 247 (21.5%) | 5 (25.0%) | <0.001 |
| Malignancy | 1010 (4.9%) | 192 (4.5%) | 166 (4.4%) | 119 (6.4%) | 522 (4.9%) | 11 (4.7%) | 0.013 |
| Immunosuppression | 81 (0.4%) | 13 (0.3%) | 13 (0.3%) | 10 (0.5%) | 44 (0.4%) | 0.0 (0%) | 0.108 |
| ***Clinical presentation*** | | | | | | | |
| Myocardial infarction | 498 (2.4%) | 87 (2.1%) | 96 (2.6%) | 68 (3.7%) | 242 (2.3%) | 5 (2.1%) | 0.003 |
| Myocarditis | 30 (0.1%) | 5 (0.1%) | 0.0 (0%) | 5 (0.3%) | 17 (0.2%) | 0.0 (0%) | 0.132 |
| Severe pulmonary embolism | 45 (0.2%) | 8 (0.2%) | 16 (0.4%) | 0.0 (0%) | 18 (0.2%) | 0.0 (0%) | <0.001 |
| Acute renal failure | 2516 (12.2%) | 356 (8.4%) | 617 (16.4%) | 288 (15.6%) | 1229 (11.6%) | 26 (11.1%) | <0.001 |
| Acute stroke | 2059 (9.9%) | 365 (8.6%) | 504 (13.4%) | 208 (11.3%) | 962 (9.1%) | 20 (8.6%) | <0.001 |
| Ischemic | 1803 (8.7%) | 318 (7.5%) | 422 (11.2%) | 183 (9.9%) | 862 (8.1%) | 18 (7.7%) | <0.001 |
| Hemorrhagic | 358 (1.7%) | 59 (1.4%) | 111 (2.9%) | 34 (1.8%) | 152 (1.4%) | 0.0 (0%) | <0.001 |
| Sepsis | 3631 (17.5%) | 207 (4.9%) | 1095 (29.1%) | 504 (27.3%) | 1794 (16.9%) | 31 (13.3%) | <0.001 |
| Septic shock | 174 (0.8%) | 16 (0.4%) | 44 (1.2%) | 33 (1.8%) | 81 (0.8%) | 0.0 (0%) | <0.001 |
| ***Microbiology*** | | | | | | | |
| Streptococcus | 4238 (20.5%) | 4238 (100%) | 0.0 (0%) | 0.0 (0%) | 0.0 (0%) | 0.0 (0%) | <0.001 |
| Staphylococcus | 3770 (18.2%) | 0.0 (0%) | 3770 (100%) | 0.0 (0%) | 0.0 (0%) | 0.0 (0%) | <0.001 |
| Gram-negative | 1849 (8.9%) | 0.0 (0%) | 0.0 (0%) | 1849 (100%) | 0.0 (0%) | 0.0 (0%) | <0.001 |
| BCNIE | 10625 (51.2%) | 0.0 (0%) | 0.0 (0%) | 0.0 (0%) | 10625 (100%) | 0.0 (0%) | <0.001 |
| Others | 234 (1.1%) | 0.0 (0%) | 0.0 (0%) | 0.0 (0%) | 0.0 (0%) | 234 (100%) | <0.001 |
| ***Treatments*** | | | | | | | |
| Transoesophageal echocardiography | 13743 (66.3%) | 2958 (69.8%) | 2664 (70.6%) | 1338 (72.3%) | 6616 (62.2%) | 167 (71.3%) | <0.001 |
| Invasive ventilation | 3090 (14.9%) | 504 (11.9%) | 705 (18.7%) | 350 (18.9%) | 1502 (14.1%) | 29 (12.4%) | <0.001 |
| Non-invasive ventilation | 827 (4.0%) | 141 (3.3%) | 193 (5.1%) | 99 (5.4%) | 389 (3.7%) | 5 (2.1%) | <0.001 |
| Dialysis | 2957 (14.3%) | 478 (11.3%) | 734 (19.5%) | 334 (18.1%) | 1383 (13.0%) | 28 (12.0%) | <0.001 |
| Transfusion | 8202 (39.6%) | 1645 (38.8%) | 1788 (47.4%) | 885 (47.9%) | 3795 (35.7%) | 89 (38.0%) | <0.001 |
| Valve surgery - mechanical prothesis | 1186 (5.7%) | 336 (7.9%) | 261 (6.9%) | 80 (4.3%) | 503 (4.7%) | 6 (2.6%) | <0.001 |
| Valve surgery - biological prothesis | 2587 (12.5%) | 570 (13.5%) | 550 (14.6%) | 232 (12.6%) | 1204 (11.3%) | 31 (13.3%) | <0.001 |
| ***Outcomes and discharge/referral structure*** | | | | | | | |
| In-hospital mortality | 2784 (13.4%) | 372 (8.8%) | 605 (16.1%) | 287 (15.5%) | 1500 (14.1%) | 20 (8.6%) | <0.001 |
| Discharge to another hospital | 6358 (30.7%) | 1309 (30.9%) | 1272 (33.7%) | 441 (23.9%) | 3270 (30.8%) | 66 (28.2%) | <0.001 |
| Discharge to rehabilitation | 2255 (10.9%) | 503 (11.9%) | 441 (11.7%) | 183 (9.9%) | 1107 (10.4%) | 21 (9.0%) | 0.018 |
| Discharge to nursing home | 271 (1.3%) | 44 (1.0%) | 48 (1.3%) | 59 (3.2%) | 114 (1.1%) | 6 (2.6%) | <0.001 |
| Discharge others | 9046 (43.7%) | 2010 (47.4%) | 1404 (37.2%) | 879 (47.5%) | 4632 (43.6%) | 121 (51.7%) | <0.001 |

Abbreviations: BCNIE, blood culture-negative infective endocarditis; COPD, chronic obstructive pulmonary disease; PAH, pulmonary arterial hypertension.

**Supplementary Table S4.** Baseline characteristics in patients with infective endocarditis and stratified by microbiology between 2011 and 2013

| **Variables** | **2011-2013** | **Streptococcus** | **Staphylococcus** | **Gram-negative** | **BCNIE** | **Others** | **p-value** |
| --- | --- | --- | --- | --- | --- | --- | --- |
|  | **(n=18446)** | **(n=3738)** | **(n=3772)** | **(n=2102)** | **(n=8690)** | **(n=144)** |  |
| ***Demographics*** | | | | | | | |
| Age (years) | 66.2 ± 14.8 | 65.7 ± 14.6 | 65.1 ± 15.4 | 69.6 ± 13.5 | 66.1 ± 14.8 | 65.0 ± 15.0 | <0.001 |
| Sex, female | 6057 (32.8%) | 896 (24.0%) | 1260 (33.4%) | 898 (42.7%) | 2961 (34.1%) | 42 (29.2%) | <0.001 |
| Length of hospital stay | 23.5 ± 17.9 | 24.3 ± 15.8 | 25.0 ± 18.5 | 32.3 ± 23.5 | 20.4 ± 16.0 | 25.1 ± 17.2 | <0.001 |
| ***Comorbidities*** | | | | | | | |
| Hypertension | 8209 (44.5%) | 1700 (45.5%) | 1645 (43.6%) | 1000 (47.6%) | 3801 (43.7%) | 63 (43.8%) | 0.012 |
| Diabetes | 4981 (27.0%) | 879 (23.5%) | 1117 (29.6%) | 744 (35.4%) | 2209 (25.4%) | 32 (22.2%) | <0.001 |
| COPD | 1555 (8.4%) | 281 (7.5%) | 344 (9.1%) | 222 (10.6%) | 698 (8.0%) | 10 (6.9%) | <0.001 |
| PAH | 1392 (7.6%) | 237 (6.3%) | 346 (9.2%) | 194 (9.2%) | 605 (7.0%) | 10 (6.9%) | <0.001 |
| History of Stroke | 1422 (7.7%) | 245 (6.6%) | 383 (10.2%) | 181 (8.6%) | 605 (7.0%) | 8 (5.6%) | <0.001 |
| Atrial fibrillation | 6669 (36.2%) | 1257 (33.6%) | 1459 (38.7%) | 903 (43.0%) | 2993 (34.4%) | 57 (39.6%) | <0.001 |
| Hypercholesterinemia | 3850 (20.9%) | 829 (22.2%) | 760 (20.2%) | 499 (23.7%) | 1735 (20.0%) | 27 (18.8%) | <0.001 |
| Peripheral vascular disease | 1051 (5.7%) | 151 (4.0%) | 294 (7.8%) | 178 (8.5%) | 423 (4.9%) | 5 (3.5%) | <0.001 |
| Chronic kidney disease | 6378 (34.6%) | 1121 (30.0%) | 1466 (38.9%) | 885 (42.1%) | 2863 (33.0%) | 43 (30.0%) | <0.001 |
| Heart failure | 6810 (36.9%) | 1298 (34.7%) | 1481 (39.3%) | 918 (43.7%) | 3066 (35.3%) | 47 (32.6%) | <0.001 |
| History of bypass surgery | 1513 (8.2%) | 306 (8.2%) | 333 (8.8%) | 182 (8.7%) | 680 (7.8%) | 12 (8.3%) | 0.381 |
| Cardiac valve status |  |  |  |  |  |  |  |
| Mechanical prothesis | 2047 (11.1%) | 415 (11.1%) | 380 (10.1%) | 233 (11.1%) | 993 (11.4%) | 26 (18.1%) | 0.017 |
| Biological prothesis | 2820 (15.3%) | 664 (17.8%) | 530 (14.1%) | 353 (16.8%) | 1253 (14.4%) | 20 (13.9%) | <0.001 |
| Native valve | 13707 (74.3%) | 2693 (72.0%) | 2892 (76.7%) | 1534 (73.0%) | 6488 (75.0%) | 100 (69.4%) | <0.001 |
| Implanted pacing device | 2615 (14.2%) | 432 (11.6%) | 658 (17.4%) | 300 (14.3%) | 1206 (13.9%) | 19 (13.2%) | <0.001 |
| Change or extraction of pacing device | 638 (24.3%) | 77 (17.8%) | 220 (33.4%) | 80 (26.6%) | 256 (21.2%) | 5 (26.3%) | <0.001 |
| Malignancy | 897 (4.9%) | 169 (4.5%) | 201 (5.3%) | 131 (6.2%) | 389 (4.5%) | 7 (4.9%) | 0.007 |
| Immunosuppression | 61 (0.3%) | 13 (0.4%) | 17 (0.5%) | 6 (0.3%) | 25 (0.3%) | 0.0 (0%) | 0.596 |
| ***Clinical presentation*** | | | | | | | |
| Myocardial infarction | 507 (2.8%) | 81 (2.2%) | 107 (2.8%) | 66 (3.1%) | 250 (2.9%) | 0.0 (0%) | <0.001 |
| Myocarditis | 20 (0.1%) | 0.0 (0%) | 0.0 (0%) | 0.0 (0%) | 0.0 (0%) | 0.0 (0%) | 0.132 |
| Severe pulmonary embolism | 48 (0.3%) | 0.0 (0%) | 15 (0.4%) | 6 (0.3%) | 23 (0.3%) | 0.0 (0%) | <0.001 |
| Acute renal failure | 2777 (15.1%) | 446 (11.9%) | 762 (20.2%) | 433 (20.6%) | 1119 (12.9%) | 17 (11.8%) | <0.001 |
| Stroke | 1999 (10.8%) | 356 (9.5%) | 512 (13.6%) | 278 (13.2%) | 842 (9.7%) | 11 (7.6%) | <0.001 |
| Ischemic | 1752 (9.5%) | 311 (8.3%) | 451 (12.0%) | 237 (11.3%) | 742 (8.5%) | 11 (7.6%) | <0.001 |
| Hemorrhagic | 351 (1.9%) | 62 (1.7%) | 95 (2.5%) | 59 (2.8%) | 135 (1.6%) | 0.0 (0%) | <0.001 |
| Sepsis | 3578 (19.4%) | 241 (6.5%) | 1148 (30.4%) | 644 (30.6%) | 1515 (17.4%) | 30 (20.8%) | <0.001 |
| Septic shock | 836 (4.5%) | 108 (2.9%) | 247 (6.6%) | 131 (6.2%) | 344 (4.0%) | 6 (4.2%) | <0.001 |
| ***Microbiology*** | | | | | | | |
| Streptococcus | 3738 (20.3%) | 3738 (100%) | 0.0 (0%) | 0.0 (0%) | 0.0 (0%) | 0.0(0%) | <0.001 |
| Staphylococcus | 3772 (20.4%) | 0.0 (0%) | 3772 (100%) | 0.0 (0%) | 0.0 (0%) | 0.0 (0%) | <0.001 |
| Gram-negative | 2102 (11.4%) | 0.0 (0%) | 0.0 (0%) | 2102 (100%) | 0.0 (0%) | 0.0 (0%) | <0.001 |
| BCNIE | 8690 (47.1%) | 0.0 (0%) | 0.0 (0%) | 0.0 (0%) | 8690 (100%) | 0.0 (0%) | <0.001 |
| Others | 144 (0.8%) | 0.0 (0%) | 0.0 (0%) | 0.0 (0%) | 0.0 (0%) | 144 (100%) | <0.001 |
| ***Treatments*** | | | | | | | |
| Transoesophageal echocardiography | 13402 (72.6%) | 2845 (76.1%) | 2850 (75.5%) | 1642 (78.1%) | 5947 (68.4%) | 118 (81.9% | <0.001 |
| Invasive ventilation | 2969 (16.1%) | 492 (13.2%) | 779 (20.7%) | 444 (21.1%) | 1244 (14.3%) | 10 (6.9%) | <0.001 |
| Non-invasive ventilation | 1086 (5.9%) | 208 (5.6%) | 279 (7.4%) | 177 (8.4%) | 418 (4.8%) | 4 (2.8%) | <0.001 |
| Dialysis | 2803 (15.2%) | 415 (11.1%) | 761 (20.2%) | 458 (21.8%) | 1151 (13.3%) | 18 (12.5%) | <0.001 |
| Transfusion | 7781 (42.2%) | 1585 (42.4%) | 1909 (50.6%) | 1016 (48.3%) | 3218 (37.0%) | 53 (36.8%) | <0.001 |
| Valve surgery - mechanical prothesis | 759 (4.1%) | 224 (6.0%) | 159 (4.2%) | 64 (3.0%) | 308 (3.5%) | 4 (2.8%) | <0.001 |
| Valve surgery - biological prothesis | 2898 (15.7%) | 669 (17.9%) | 730 (19.4%) | 320 (15.2%) | 1159 (13.3%) | 20 (13.9%) | <0.001 |
| ***Outcomes and discharge/referral structure*** | | | | | | | |
| In-hospital mortality | 2629 (14.3%) | 329 (8.8%) | 670 (17.8%) | 371 (17.7%) | 1249 (14.4%) | 10 (6.9%) | <0.001 |
| Discharge to another hospital | 6073 (32.9%) | 1245 (33.3%) | 1314 (34.8%) | 584 (27.8%) | 2884 (33.2%) | 46 (31.9%) | <0.001 |
| Discharge to rehabilitation | 1940 (10.5%) | 482 (12.9%) | 397 (10.5%) | 174 (8.3%) | 872 (10.0%) | 15 (10.4%) | <0.001 |
| Discharge to nursing home | 263 (1.4%) | 43 (1.2%) | 50 (1.3%) | 46 (2.2%) | 122 (1.4%) | 0.0 (0%) | <0.001 |
| Discharge others | 7541 (40.9%) | 1639 (43.9%) | 1341 (35.6%) | 927 (44.1%) | 3563 (41.0%) | 71 (49.3%) | <0.001 |

Abbreviations: BCNIE, blood culture-negative infective endocarditis; COPD, chronic obstructive pulmonary disease; PAH, pulmonary arterial hypertension.**Supplementary Table S5.** Baseline characteristics in patients with infective endocarditis and stratified by microbiology between 2014 and 2016

| **Variables** | **2014-2016** | **Streptococcus** | **Staphylococcus** | **Gram-negative** | **BCNIE** | **Others** | **p-value** |
| --- | --- | --- | --- | --- | --- | --- | --- |
|  | **(n=21961)** | **(n=4358)** | **(n=4695)** | **(n=2992)** | **(n=9704)** | **(n=212)** |  |
| ***Demographics*** | | | | | | | |
| Age (years) | 66.9 ± 14.7 | 67.1 ± 14.45 | 65.9 ± 15.4 | 69.8 ± 13.2 | 66.5 ± 14.9 | 65.3 ± 16.0 | <0.001 |
| Sex, female | 6986 (31.8%) | 1108 (25.4%) | 1439 (30.7%) | 1196 (40.0%) | 3182 (32.8%) | 61 (28.8%) | <0.001 |
| Length of hospital stay | 24.2 ± 18.7 | 24.9 ± 16.2 | 25.2 ± 18.4 | 33.4 ± 25.9 | 20.6 ± 16.1 | 27.2 ± 18.9 | <0.001 |
| ***Comorbidities*** | | | | | | | |
| Hypertension | 10117 (46.1%) | 2089 (47.9%) | 2134 (45.6%) | 1427 (47.7%) | 4368 (45.0%) | 99 (46.7%) | <0.001 |
| Diabetes | 6188 (28.2%) | 1071 (24.6%) | 1405 (29.9%) | 1123 (37.5%) | 2537 (26.1%) | 52 (24.5%) | <0.001 |
| COPD | 2041 (9.3%) | 353 (8.1%) | 426 (9.1%) | 370 (12.4%) | 872 (9.0%) | 20 (9.4%) | <0.001 |
| PAH | 1988 (9.1%) | 393 (9.0%) | 469 (10.0%) | 327 (10.9%) | 773 (8.0%) | 26 (12.3%) | <0.001 |
| History of Stroke | 1886 (8.6%) | 334 (7.7%) | 475 (10.1%) | 291 (9.7%) | 766 (7.9%) | 20 (9.4%) | <0.001 |
| Atrial fibrillation | 8828 (40.2%) | 1728 (39.7%) | 1929 (41.1%) | 1392 (46.5%) | 3698 (38.1%) | 81 (38.2%) | <0.001 |
| Hypercholesterinemia | 5217 (23.8%) | 1095 (25.1%) | 1118 (23.8%) | 758 (25.3%) | 2197 (22.6%) | 49 (23.1%) | 0.004 |
| Peripheral vascular disease | 1389 (6.3%) | 203 (4.7%) | 379 (8.1%) | 289 (9.7%) | 508 (5.2%) | 10 (4.7%) | <0.001 |
| Chronic kidney disease | 7636 (34.8%) | 1339 (30.7%) | 1705 (36.3%) | 1306 (43.7%) | 3221 (33.2%) | 65 (30.7%) | <0.001 |
| Heart failure | 9154 (41.7%) | 1644 (37.7%) | 2043 (43.5%) | 1498 (50.1%) | 3871 (39.9%) | 98 (46.2%) | <0.001 |
| History of bypass surgery | 1961 (8.9%) | 410 (9.4%) | 440 (9.4%) | 305 (10.2%) | 791 (8.2%) | 15 (7.1%) | 0.003 |
| Cardiac valve status |  |  |  |  |  |  |  |
| Mechanical prothesis | 2215 (10.1%) | 466 (10.7%) | 455 (9.7%) | 299 (10.0%) | 962 (9.9%) | 33 (15.6%) | 0.041 |
| Biological prothesis | 4275 (19.5%) | 976 (22.4%) | 813 (17.3%) | 608 (20.3%) | 1840 (19.0%) | 38 (17.9%) | <0.001 |
| Native valve | 15625 (71.2%) | 2947 (67.6%) | 3467 (73.8%) | 2117 (70.8%) | 6950 (71.6%) | 144 (67.9%) | <0.001 |
| Implanted pacing device | 3696 (16.8%) | 633 (14.5%) | 924 (19.7%) | 569 (19.0%) | 1535 (15.8%) | 35 (16.5%) | <0.001 |
| Change or extraction of pacing device | 1056 (28.5%) | 120 (18.9%) | 380 (41.1%) | 164 (28.8%) | 377 (24.5%) | 15 (42.8%) | <0.001 |
| Malignancy | 1120 (5.1%) | 186 (4.3%) | 237 (5.1%) | 181 (6.1%) | 503 (5.2%) | 13 (6.1%) | 0.014 |
| Immunosuppression | 83 (0.4%) | 10 (0.2%) | 18 (0.4%) | 23 (0.8%) | 31 (0.3%) | 0.0 (0%) | <0.001 |
| ***Clinical presentation*** | | | | | | | |
| Myocardial infarction | 608 (2.8%) | 81 (1.9%) | 144 (3.1%) | 97 (3.2%) | 282 (2.9%) | 4 (1.9%) | 0.001 |
| Myocarditis | 31 (0.1%) | 0.0 (0%) | 8 (0.2%) | 7 (0.2%) | 6 (0.2%) | 0.0 (0%) | <0.001 |
| Severe pulmonary embolism | 71 (0.3%) | 3 (0.1%) | 18 (0.4%) | 18 (0.6%) | 32 (0.3%) | 0.0 (0%) | 0.002 |
| Acute renal failure | 5008 (22.8%) | 767 (17.6%) | 1371 (29.2%) | 961 (32.1%) | 1861 (19.2%) | 48 (22.6%) | <0.001 |
| Stroke | 2589 (11.8%) | 476 (10.9%) | 654 (13.9%) | 397 (13.3%) | 1037 (10.7%) | 25 (11.8%) | <0.001 |
| Ischemic | 2312 (10.5%) | 420 (9.6%) | 574 (12.2%) | 362 (12.1%) | 934 (9.6%) | 22 (10.4%) | <0.001 |
| Hemorrhagic | 445 (2.0%) | 79 (1.8%) | 141 (3.0%) | 65 (2.2%) | 156 (1.6%) | 4 (1.9%) | <0.001 |
| Sepsis | 4759 (21.7%) | 278 (6.4%) | 1600 (34.1%) | 1002 (33.5%) | 1824 (18.8%) | 55 (25.9%) | <0.001 |
| Septic shock | 1265 (5.8%) | 134 (3.1%) | 367 (7.8%) | 267 (8.9%) | 491 (5.1%) | 6 (2.8%) | <0.001 |
| ***Microbiology*** | | | | | | | |
| Streptococcus | 4358 (19.8%) | 4358 (100%) | 0.0 (0%) | 0.0 (0%) | 0.0 (0%) | 0.0 (0%) | <0.001 |
| Staphylococcus | 4695 (21.4%) | 0.0 (0%) | 4695 (100%) | 0.0 (0%) | 0.0 (0%) | 0.0 (0%) | <0.001 |
| Gram-negative | 2992 (13.6%) | 0.0 (0%) | 0.0 (0%) | 2992 (100%) | 0.0 (0%) | 0.0 (0%) | <0.001 |
| BCNIE | 9704 (44.2%) | 0.0 (0%) | 0.0 (0%) | 0.0 (0%) | 9704 (100%) | 0.0 (0%) | <0.001 |
| Others | 212 (1.0%) | 0.0 (0%) | 0.0 (0%) | 0.0 (0%) | 0.0 (0%) | 212 (100%) | <0.001 |
| ***Treatments*** | | | | | | | |
| Transoesophageal echocardiography | 16681 (75.9%) | 3484 (79.9%) | 3663 (78.0%) | 2459 (82.1%) | 6917 (71.2%) | 158 (74.5%) | <0.001 |
| Invasive ventilation | 3844 (17.5%) | 592 (13.6%) | 1035 (22.0%) | 710 (23.7%) | 1470 (15.2%) | 37 (17.5%) | <0.001 |
| Non-invasive ventilation | 1700 (7.7%) | 294 (6.8%) | 433 (9.2%) | 361 (12.1%) | 596 (6.1%) | 16 (7.6%) | <0.001 |
| Dialysis | 3284 (15.0%) | 407 (9.3%) | 926 (19.7%) | 650 (21.7%) | 1274 (13.1%) | 27 (12.7%) | <0.001 |
| Transfusion | 8921 (40.6%) | 1745 (40.0%) | 2253 (48.0%) | 1438 (48.0%) | 3390 (34.9%) | 95 (44.8%) | <0.001 |
| Valve surgery - mechanical prothesis | 760 (3.5%) | 224 (5.1%) | 175 (3.7%) | 68 (2.3%) | 283 (2.9%) | 10 (4.7%) | <0.001 |
| Valve surgery - biological prothesis | 3633 (16.5%) | 792 (18.2%) | 926 (19.7%) | 506 (16.9%) | 1369 (14.1%) | 40 (18.9%) | <0.001 |
| ***Outcomes and discharge/referral structure*** | | | | | | | |
| In-hospital mortality | 3253 (14.81) | 360 (8.3%) | 898 (19.1%) | 561 (18.8%) | 1408 (14.5%) | 26 (12.3%) | <0.001 |
| Discharge to another hospital | 7470 (34.0%) | 1589 (36.5%) | 1643 (35.0%) | 878 (29.3%) | 3285 (33.9%) | 75 (35.4%) | <0.001 |
| Discharge to rehabilitation | 2148 (9.8%) | 488 (11.2%) | 440 (9.4%) | 311 (10.4%) | 885 (9.1%) | 24 (11.3%) | 0.002 |
| Discharge to nursing home | 333 (1.5%) | 54 (1.2%) | 75 (1.6%) | 71 (2.4%) | 130 (1.3%) | 3 (1.4%) | 0.001 |
| Discharge others | 8757 (39.9%) | 1867 (42.8%) | 1639 (34.9%) | 1171 (39.1%) | 3996 (41.2%) | 84 (39.6%) | <0.001 |

Abbreviations: BCNIE, blood culture-negative infective endocarditis; COPD, chronic obstructive pulmonary disease; PAH, pulmonary arterial hypertension.

**Supplementary Table S6.** Baseline characteristics in patients with infective endocarditis and stratified by microbiology between 2017 and 2019

| **Variables** | **2017-2019** | **Streptococcus** | **Staphylococcus** | **Gram-negative** | **BCNIE** | **Others** | **p-value** |
| --- | --- | --- | --- | --- | --- | --- | --- |
|  | **(n=25346)** | **(n=5284)** | **(n=5436)** | **(n=3923)** | **(n=10390)** | **(n=313)** |  |
| ***Demographics*** | | | | | | | |
| Age (years) | 67.6 ± 14.5 | 67.6 ± 14.2 | 66.5 ± 15.4 | 70.4 ± 13.3 | 67.2 ± 14.5 | 65.8 ± 15.0 | <0.001 |
| Sex, female | 7584 (29.9%) | 1148 (21.7%) | 1663 (30.6%) | 1511 (38.5%) | 3178 (30.6%) | 84 (26.8%) | <0.001 |
| Length of hospital stay | 23.6 ± 17.8 | 24.4 ± 16.5 | 24.2 ± 17.6 | 30.9 ± 22.4 | 20.0 ± 15.4 | 26.8 ± 19.5 | <0.001 |
| ***Comorbidities*** | | | | | | | |
| Hypertension | 12250 (48.3%) | 2592 (49.1%) | 2528 (46.5%) | 1959 (49.9%) | 5027 (48.4%) | 144 (46.0%) | 0.011 |
| Diabetes | 7161 (28.3%) | 1325 (25.1%) | 1652 (30.4%) | 1374 (35.0%) | 2735 (26.3%) | 75 (24.0%) | <0.001 |
| COPD | 2342 (9.2%) | 450 (8.5%) | 507 (9.3%) | 450 (11.5%) | 911 (8.8%) | 24 (7.7%) | <0.001 |
| PAH | 2129 (8.4%) | 481 (9.1%) | 455 (8.4%) | 392 (10.0%) | 774 (7.5%) | 27 (8.6%) | <0.001 |
| History of Stroke | 2298 (9.0%) | 420 (8.0%) | 620 (11.4%) | 395 (10.1%) | 833 (8.0%) | 30 (9.6%) | <0.001 |
| Atrial fibrillation | 10833 (42.7%) | 2278 (43.1%) | 2294 (42.2%) | 1960 (50.0%) | 4167 (40.1%) | 134 (42.8%) | <0.001 |
| Hypercholesterinemia | 6814 (26.9%) | 1477 (28.0%) | 1348 (24.8%) | 1153 (29.4%) | 2743 (26.4%) | 93 (29.7%) | <0.001 |
| Peripheral vascular disease | 1708 (6.7%) | 269 (5.1%) | 426 (7.8%) | 408 (10.4%) | 582 (5.6%) | 23 (7.4%) | <0.001 |
| Chronic kidney disease | 8636 (34.1%) | 1576 (29.8%) | 1870 (34.4%) | 1684 (42.9%) | 3414 (32.9%) | 92 (29.4%) | <0.001 |
| Heart failure | 11444 (45.2%) | 2293 (43.4%) | 2528 (46.5%) | 2053 (52.3%) | 4412 (42.5%) | 158 (50.5%) | <0.001 |
| History of bypass surgery | 2143 (8.5%) | 465 (8.8%) | 471 (8.7%) | 369 (9.4%) | 813 (7.8%) | 25 (8.0%) | 0.025 |
| Cardiac valve status |  |  |  |  |  |  |  |
| Mechanical prothesis | 2297 (9.0%) | 482 (9.1%) | 421 (7.7%) | 341 (8.7%) | 1015 (9.8%) | 38 (12.1%) | <0.001 |
| Biological prothesis | 5449 (21.5%) | 1334 (25.3%) | 995 (18.3%) | 843 (21.5%) | 2217 (21.3%) | 60 (19.2%) | <0.001 |
| Native valve | 17776 (70.1%) | 3507 (66.4%) | 4048 (74.5%) | 2776 (70.8%) | 7228 (69.6%) | 217 (69.3%) | <0.001 |
| Implanted pacing device | 4319 (17.0%) | 826 (15.6%) | 995 (18.3%) | 749 (19.1%) | 1708 (16.4%) | 41 (13.1%) | <0.001 |
| Change or extraction of pacing device | 1110 (25.7%) | 147 (17.7%) | 370 (37.1%) | 209 (27.9%) | 368 (21.5%) | 16 (39.0%) | <0.001 |
| Malignancy | 1262 (5.0%) | 204 (3.9%) | 287 (5.3%) | 201 (5.1%) | 558 (5.4%) | 12 (3.8%) | <0.001 |
| Immunosuppression | 78 (0.3%) | 17 (0.3%) | 12 (0.2%) | 14 (0.4%) | 33 (0.3%) | 0.0 (0%) | <0.001 |
| ***Clinical presentation*** | | | | | | | |
| Myocardial infarction | 699 (2.8%) | 115 (2.2%) | 146 (2.7%) | 144 (3.7%) | 289 (2.8%) | 0.0 (0%) | <0.001 |
| Myocarditis | 34 (0.1%) | 6 (0.1%) | 5 (0.1%) | 8 (0.2%) | 15 (0.1%) | 0.0 (0%) | 0.589 |
| Severe pulmonary embolism | 85 (0.3%) | 12 (0.2%) | 19 (0.4%) | 10 (0.3%) | 42 (0.4%) | 0.0 (0%) | <0.001 |
| Acute renal failure | 7399 (29.2%) | 1228 (23.2%) | 1968 (36.2%) | 1510 (38.5%) | 2595 (25.0%) | 98 (31.3%) | <0.001 |
| Stroke | 3163 (12.5%) | 588 (11.1 %) | 818 (15.1%) | 560 (14.3%) | 1156 (11.1%) | 41 (13.1%) | <0.001 |
| Ischemic | 2846 (11.2%) | 521 (9.9%) | 729 (13.4%) | 510 (13.0%) | 1049 (10.1%) | 37 (11.8%) | <0.001 |
| Hemorrhagic | 533 (2.1%) | 99 (1.9%) | 165 (3.0%) | 83 (2.1%) | 175 (1.7%) | 11 (3.5%) | <0.001 |
| Sepsis | 5495 (21.7%) | 339 (6.4%) | 1860 (34.2%) | 1313 (33.5%) | 1906 (18.3%) | 77 (24.6%) | <0.001 |
| Septic shock | 1652 (6.5%) | 185 (3.5%) | 496 (9.1%) | 374 (9.5%) | 572 (5.5%) | 25 (8.0%) | <0.001 |
| ***Microbiology*** | | | | | | | |
| Streptococcus | 5284 (20.8%) | 5284 (100%) | 0.0 (0%) | 0.0 (0%) | 0.0 (0%) | 0.0 (0%) | <0.001 |
| Staphylococcus | 5436 (21.4%) | 0.0 (0%) | 5436 (100%) | 0.0 (0%) | 0.0 (0%) | 0.0 (0%) | <0.001 |
| Gram-negative | 3923 (15.5%) | 0.0 (0%) | 0.0 (0%) | 3923 (100%) | 0.0 (0%) | 0.0 (0%) | <0.001 |
| BCNIE | 10390 (41.0%) | 0.0 (0%) | 0.0 (0%) | 0.0 (0%) | 10390 (100%) | 0.0 (0%) | <0.001 |
| Others | 313 (1.2%) | 0.0 (0%) | 0.0 (0%) | 0.0 (0%) | 0.0 (0%) | 313 (100%) | <0.001 |
| ***Treatments*** | | | | | | | |
| Transoesophageal echocardiography | 19412 (76.5%) | 4183 (79.1%) | 4258 (78.3%) | 3160 (80.5%) | 7574 (72.9 %) | 237 (75.72 %) | <0.001 |
| Invasive ventilation | 4329 (17.1%) | 724 (13.7%) | 1115 (20.5%) | 955 (24.3%) | 1474 (14.2%) | 61 (19.5%) | <0.001 |
| Non-invasive ventilation | 2051 (8.1%) | 362 (6.9%) | 531 (9.8%) | 467 (11.9%) | 651 (6.3%) | 40 (12.8%) | <0.001 |
| Dialysis | 3783 (14.9%) | 522 (9.9%) | 1089 (20.0%) | 844 (21.5%) | 1270 (12.2%) | 58 (18.5%) | <0.001 |
| Transfusion | 10099 (39.8%) | 2086 (39.5%) | 2510 (46.2%) | 1877 (47.9%) | 3463 (33.3%) | 163 (52.1%) | <0.001 |
| Valve surgery - mechanical prothesis | 641 (2.5%) | 208 (3.9%) | 138 (2.5%) | 61 (1.6%) | 210 (2.0%) | 24 (7.7%) | <0.001 |
| Valve surgery - biological prothesis | 4476 (17.7%) | 1066 (20.2%) | 1086 (20.0%) | 763 (19.5%) | 1489 (14.3%) | 72 (23.0%) | <0.001 |
| ***Outcomes and discharge/referral structure*** | | | | | | | |
| In-hospital mortality | 4186 (16.5%) | 557 (10.5%) | 1098 (20.2%) | 817 (20.8%) | 1660 (16.0%) | 54 (17.2%) | <0.001 |
| Discharge to another hospital | 8900 (35.1%) | 1956 (37.0%) | 2018 (37.1%) | 1209 (30.8%) | 3612 (34.8%) | 105 (33.6%) | <0.001 |
| Discharge to rehabilitation | 2093 (8.3%) | 553 (10.5%) | 385 (7.1%) | 318 (8.1%) | 810 (7.8%) | 27 (8.6%) | <0.001 |
| Discharge to nursing home | 400 (1.6%) | 72 (1.4%) | 70 (1.3%) | 111 (2.8%) | 140 (1.4%) | 7 (2.2%) | <0.001 |
| Discharge others | 9767 (38.5%) | 2146 (40.6%) | 1865 (34.3%) | 1468 (37.4%) | 4168 (40.1%) | 120 (38.3%) | <0.001 |

Abbreviations: BCNIE, blood culture-negative infective endocarditis; COPD, chronic obstructive pulmonary disease; PAH, pulmonary arterial hypertension.
